# Long-Term Spatio-Temporal Trends of Organotin Contaminations in the Marine Environment of Hong Kong

**DOI:** 10.1371/journal.pone.0155632

**Published:** 2016-05-13

**Authors:** Kevin K. Y. Ho, Guang-Jie Zhou, Elvis G. B. Xu, Xinhong Wang, Kenneth M. Y. Leung

**Affiliations:** 1 The Swire Institute of Marine Science and School of Biological Sciences, The University of Hong Kong, Pokfulam, Hong Kong, China; 2 State Key Laboratory of Marine Environmental Science (Xiamen University), Xiamen University, Xiamen, China; 3 The State Key Laboratory in Marine Pollution, City University of Hong Kong, Tat Chee Avenue, Kowloon, Hong Kong, China; Seoul National University, REPUBLIC OF KOREA

## Abstract

Hong Kong imposed a partial restriction on application of organotin-based antifouling paints in 1992. Since September 2008, the International Maritime Organization prohibited the use of such antifouling systems on all sea-going vessels globally. Therefore, it is anticipated a gradual reduction of organotin contamination in Hong Kong’s marine waters. Using the rock shell *Reishia clavigera* as a biomonitor, we evaluated the organotin contamination along Hong Kong’s coastal waters over the past two decades (1990–2015). In 2010 and 2015, adult *R*. *clavigera* were examined for imposex status and analysed for tissue concentrations of six organotins. We consistently found 100% imposex incidence in female *R*. *clavigera* across all sites. Tissue triphenyltin (TPT) concentrations were high in most samples. A probabilistic risk assessment showed that there were over 69% of chance that local *R*. *clavigera* would be at risk due to exposure to phenyltins. Comparing with those of previous surveys (2004–2010), both imposex levels and tissue concentrations of organotins did not decline, while the ecological risks due to exposure to organotins were increasing. We also observed high concentrations of monobutyltin and TPT in seawater and sediment from locations with intense shipping activities and from stormwater or sewage discharge. Overall, organotins are still prevalent in Hong Kong’s marine waters showing that the global convention alone may be inadequate in reducing organotin contamination in a busy international port like Hong Kong. Appropriate management actions should be taken to control the use and release of organotins in Hong Kong and South China.

## Introduction

Organotin compounds (OTs), such as tri-butyltin (TBT) and tri-phenyltin (TPT), have been extensively used as active ingredients in antifouling paints on ship hulls, open-sea mariculture cages and other submerged marine infrastructures, and as pesticides since the 1960s [1]. These compounds are also toxic to non-targeted marine organisms causing growth abnormalities and reproductive failure in bivalves and gastropods [2]. The best-documented example is OT-mediated imposex (i.e., the superimposition of penis and vas deferens on females) which is found in nearly 200 neogastropod species [3]. Although the induction mechanism is yet to be fully elucidated, imposex can be developed in the dogwhelk *Nucella lapillus* after exposure to TBT at concentrations as low as 1 ng L^-1^ [4] and the pseudo-penis of female rock shell *Reishia clavigera* (= *Thais clavigera*) can be lengthened after injection with 0.1 ng g^-1^ TPT [5]. Imposex may lead to reproductive failure and mortality in female gastropods, thus causing extinction of local populations [6].

Being a major international port, Hong Kong receives with more than 348,000 vessels annually [7] together with numerous local fishing and recreational boats. Since 1988, TBT and its degradation products, i.e., mono-butyltin (MBT) and di-butyltin (DBT) have been frequently detected in water and sediment especially in areas close to shipyards and marinas [8, 9]. Phenyltin compounds (PTs), however, were seldom monitored in this region.

Owing to the adverse environmental impacts of OTs, many countries have banned OT-based antifouling paints since the mid-1980s [10]. For instance, in 2002, the European Union (EU) prohibited the use of TPT as pesticide [11] and in 2003, the European Commission Parliament adopted a regulation (No. 782/2003) which banned the use of all OT-based antifouling paints on all vessels of EU member countries [12]. These helped reduce the contaminations of OTs in European waters. Globally, the International Maritime Organization (IMO) adopted the International Convention on the Control of Harmful Anti-fouling Systems on Ships (i.e., AFS Convention) in November 2001 [13], which prohibited the application of OTs in any antifouling systems worldwide. The AFS Convention finally received enough signatories among IMO members and was entered into force on 17 September 2008 [10]. Locally, Hong Kong has banned the use of TBT on small vessels (i.e., < 25 m in length) since 1992 [14]. TPT, on the other hand, is not a registered pesticide in Hong Kong. Therefore, it is hypothesized that after the commencement of the global ban of OT-based antifouling paints, OT contamination in the local coastal waters should have been reduced gradually.

This study used the rock shell *R*. *clavigera* as a biomonitor, together with water and sediment collected from Hong Kong’s coastal waters to test the above hypothesis. This species has been used for monitoring OT contamination over the Asia-Pacific region [3] since its first description of imposex [15]. In Hong Kong, 100% of imposex incidence was first noted in 11 sampling sites in 1992 [16]. Subsequent surveys reported that over 91% of female *R*. *clavigera* developed imposex [17, 18]. Recent studies again showed 100% imposex incidence in all survey sites [14, 19, 20]. With commencement of the global prohibition of OT-based antifouling paints, this study tested the hypothesis of recovery of OT contamination through six specific objectives: (1) examination of imposex status of *R*. *clavigera* along the coast of Hong Kong in 2010 and 2015; (2) determination of tissue concentrations of BTs and, for the first time PTs, in *R*. *clavigera* in Hong Kong; (3) quantification of concentrations of OTs in water and sediment samples; (4) temporal comparisons of current imposex status and tissue concentrations of BTs in *R*. *clavigera* with data obtained from previous studies (i.e., Leung et al. [14] and Qiu et al. [20]); (5) assessment of ecological risks of total BTs and total PTs to *R*. *clavigera* populations in Hong Kong using a tissue-residue based probabilistic approach; and (6) elucidation of potential sources of OTs in Hong Kong. The results generated from this study can provide important information for indicating the ecological risks associated with OTs in this region and evaluating the effectiveness of global regulatory measures on the reduction of OT contamination in a busy international port like Hong Kong.

## Materials and Methods

### Sample collection of rock shells

**Fig 1:**
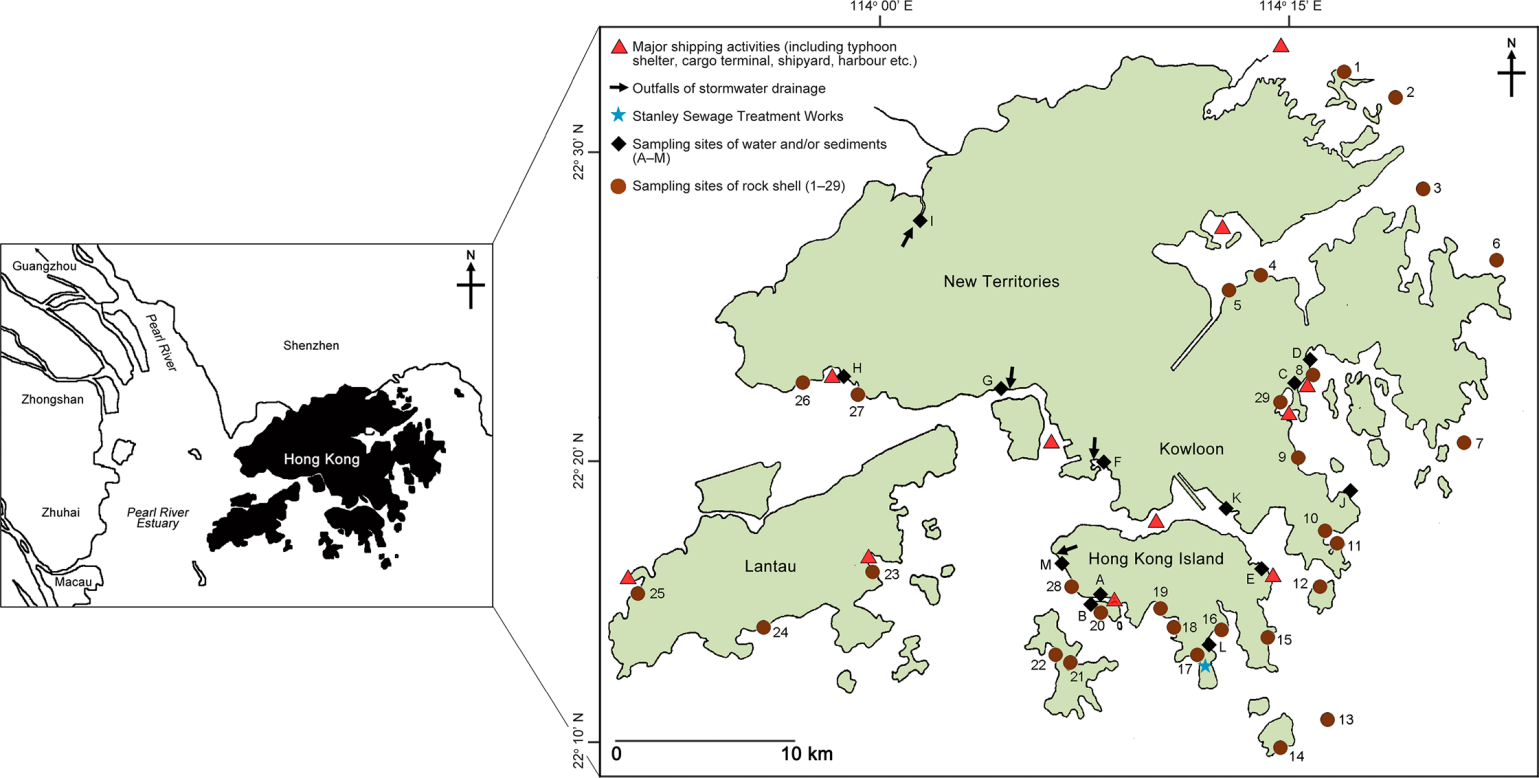
Sampling sites of seawater and/or sediments (dark diamonds), and the rock shell *Reishia clavigera* (brown circles) along the coast of Hong Kong in 2010, 2014 and 2015. Locations of the major facilities with intense marine traffic are indicated by red triangles. Arrows indicate the outfalls of stormwater drainage, and the blue star indicates the Stanley Sewage Treatment Works.

About 40 adult *R*. *clavigera* (shell length ≥ 17 mm [15]), having actual shell lengths between 23 and 36 mm, were randomly collected on each of the 29 rocky shores along the coast of Hong Kong (22°08' to 22°35' N, 113°49' to 114°31' E) during May to September 2010 (Fig 1; S1 Table with geographical coordinates of all sampling sites). These sites were visited by Leung et al. [14] and Qiu et al. [20] showing different degrees of OT contamination. No specific permissions were required for sampling in these locations because they are not protected areas and are accessible by general public. *Reishia clavigera* were present in all sites except Heng On where the natural rocky shore was turned into concrete block of Ma On Shan Waterfront Promenade. The animals were transported to the laboratory within 6 h after collection, and kept at -20°C before conducting imposex and chemical analyses. No depuration of the collected rock shells was conducted. The sampling was repeated on 10 selected shores in July and August 2015 (S2 Table with geographical coordinates of all sampling sites).

### Imposex determination

Each animal was measured for its shell length and fresh weight using vernier calipers (SPI 31-415-3, USA) and an electronic balance (Libror EB-430HU, Japan), respectively, then was cracked using a bench vice. Their reproductive features were examined using a dissecting microscope (Olympus SZH10, Japan). Penis length was determined using the scale in the microscopic lens. Vas Deferens Sequence Index (VDSI) and Relative Penis Size Index (RPSI) were evaluated (S3 Table) by the same person to ensure consistency.

### Seawater, in/effluent and sediment samples

Seawater and sediment were collected from five sampling stations which are proximal to shipping activities (Fig 1; see S4 Table for geographical coordinates of sites A to E) during January 2014. One litre of mixture of surface and sub-surface water (ca. 0.5 m below the water surface) and one kilogram of sediment were collected in duplicates using Van Dorn water sampler/glass bottle and Ekman grab, respectively. The seawater sampling was repeated in 2015, at 10 selected locations covering different potential sources of OTs. The influent and effluent of Stanley Sewage Treatment Works were also collected (S4 Table with geographical coordinates of all sampling sites). All samples were collected in two or three replicates, and were kept in ice and sent to the laboratory for analysis within 6 h of collection.

### Chemical analysis

Rock shells from each site were pooled as three or four replicates, each having 8–15 individuals. We adopted established methods for analyses for OTs ([21] for rock shell and sediment; [22] for water with slight modifications; see S5 Table). HPLC grade solvents were used (Tedia, USA). Chemical standards were purchased from Sigma-Aldrich (St. Louis, USA) and Chiron (Trondheim, Norway). Quantification of six OTs, including MBT, DBT, TBT, monophenyltin (MPT), diphenyltin (DPT) and TPT, was conducted using a gas chromatograph (GC; Agilent 6890, USA) equipped with a mass-selective detector (Agilent 5973). A DB-5MS fused silica capillary GC column (J&W Scientific Inc., USA) was used with film thickness of 0.25 mm i.d. × 30 m × 0.25 μm (more analytical parameters are available in S6 Table). Satisfactory recovery rates were obtained for the six OTs ranging from 64.6%–93.5% except DPT (S7 Table). The method was further validated by using a certified reference material [21]. A procedural blank was simultaneously analysed every batch of five samples. The detection limits were estimated at 0.2–1.5 μg kg^-1^ dry weight (dw) for tissue and sediment samples, and 0.4–2.6 ng L^-1^ for water samples (except 29.1 ng L^-1^ for MBT). All concentrations were reported without correction to the recovery rates.

Degradation indices of butyltins (BDI) and phenyltins (PDI) were calculated based on the measured tissue concentrations [23]:

BDI=([MBT]+[DBT])/[TBT]

PDI=([MPT]+[DPT])/[TPT]

### Statistical analysis

Mean VDSI, RPSI, percentage of sterile female, tissue TBT, TPT and total OTs concentrations were temporally compared among surveys in 2004 [14], 2005/06 [20], 2010 and 2015 (present study) using paired-samples *t* test (with log_10_-transformed data) or Wilcoxon signed-rank test. Correlations among tissue OTs concentrations, imposex indices and condition index (= fresh tissue weight x 100 / (fresh tissue weight + dry shell mass) [24] were tested using Spearman’s rank correlation analyses followed by sequential Bonferroni correction [25]. Relationships of the distance between the sampling site and its nearest harbour or marina (estimated using Google Map) and imposex indices, OTs concentrations and condition index were tested using Spearman’s rank correlation analyses with Bonferroni correction.

All statistical tests were conducted using SPSS Statistics 19.0 (SPSS Inc., USA) and Microsoft EXCEL 2003 (Microsoft Corporation, USA).

### Probabilistic ecological risk assessment

To evaluate the ecological risk of BTs and PTs on the populations of *R*. *clavigera*, a probabilistic risk assessment was conducted by calculating the risk quotient (RQ; see S8–S10 Tables):

$$\text{RQ}\;=\;\frac{\text{Measured tissue concentration}\;\left(\text{MTC}\right)}{\text{Predicted no effect tissue concentration}\;\left(\text{PNETC}\right)}$$

If the calculated RQ ≥ 1, the *R*. *clavigera* population is at risk due to the exposure to BTs or PTs [14]. Distributions of RQs (for BTs and PTs respectively), based on the distributions of respective MTCs and PNETCs which were fitted with log-logistic or Pareto models, were computed using Monte Carlo simulation with 10,000 iterations for 10 times. The RQ distribution was truncated as only RQ ≥ 0 was relevant. The simulations were carried out using @Risk 5.7 (Palisade Corporation, USA).

## Results

### Imposex status of rock shell

Imposex occurred in all females in both the 2010 and 2015 surveys (i.e., 100% imposex; Table 1). In 2010, the highest imposex levels were observed in rock shells from Sok Kwu Wan (VDSI = 5.73, RPSI = 37.70) and Sai Kung Pier (VDSI = 4.94, RPSI = 94.66), whereas the lowest VDSI (2.61) and RPSI (1.19) were observed in Waglan Island. In 2015, among the 10 survey sites, the highest imposex levels were still observed in individuals from Sok Kwu Wan (VDSI = 5.22, RPSI = 38.87) and Sai Kung Pier (VDSI = 4.78, RPSI = 48.00), while the lowest were in Po Toi (VDSI = 3.61, RPSI = 9.61) and Turtle Cove (VDSI = 3.74, RPSI = 8.05) respectively. Stage-1 individuals were found in four sites (Clear Water Bay, Deep Water Bay, Mui Wo and Kadoorie Beach) in 2010, while none of them were found in 2015. Conversely, stage-6 individuals were observed in only 17 sites (59%) in 2010, whereas in 2015 they were present in all of the 10 sites.

**Table 1:** Imposex indices (i.e., Vas Deferens Sequence Index, VDSI and Relative Penis Size Index, RPSI) and condition index of *Reishia clavigera* collected in Hong Kong.

		Mean VDSI			Median VDSI			% Imposex			% sterile F			RPSI			Condition Index		
Site		2004–06	2010	2015	2004–06	2010	2015	2004–06	2010	2015	2004–06	2010	2015	2004–06	2010	2015	2010	2015	Distance to shipping activities (km)
1	Kat O	3.92	4.08	N.E.	4.0	4.0	N.E.	100	100	N.E.	0.0	24.0	N.E.	1.59	9.33	N.E.	16.6	N.E.	2.7
2	Pak Sha Chau	3.57	3.14	N.E.	4.0	3.0	N.E.	100	100	N.E.	0.0	0.0	N.E.	0.37	8.14	N.E.	18.7	N.E.	4.9
3	Chek Chau	3.50	3.10	N.E.	3.5	3.0	N.E.	100	100	N.E.	0.0	0.0	N.E.	0.57	4.74	N.E.	18.3	N.E.	10.3
4	Wu Kwai Sha	2.67	3.56	N.E.	3.0	3.5	N.E.	100	100	N.E.	0.0	22.2	N.E.	0.17	6.00	N.E.	24.4	N.E.	4.3
5	Heng On	3.64	N.A.	N.E.	4.0	N.A.	N.E.	100	N.A.	N.E.	0.0	N.A.	N.E.	0.84	N.A.	N.E.	N.A.	N.E.	18.5
6	Wong Mau Chau	3.03	3.09	N.E.	3.0	3.0	N.E.	100	100	N.E.	0.0	18.2	N.E.	0.11	4.17	N.E.	15.8	N.E.	17.0
7	Kong Tau Pai	2.38	2.69	N.E.	2.0	3.0	N.E.	100	100	N.E.	0.0	0.0	N.E.	0.06	6.36	N.E.	19.0	N.E.	11.4
8	Sai Kung Pier	4.70	4.94	4.78	4.0	5.0	5.0	100	100	100	40.0	72.2	55.6	34.38	94.66	48.00	20.5	20.3	0.2
9	UST	4.17	4.55	N.E.	4.0	4.0	N.E.	100	100	N.E.	11.1	45.0	N.E.	8.85	48.60	N.E.	21.6	N.E.	2.9
10	Clear Water Bay	3.04	2.81	4.71	3.0	2.0	5.0	100	100	100	0.0	9.5	52.9	0.53	1.71	31.70	19.3	22.4	8.9
11	Shek Mei Tao	3.22	3.33	N.E.	3.0	3.0	N.E.	100	100	N.E.	0.0	12.5	N.E.	0.15	3.32	N.E.	19.4	N.E.	5.8
12	Tung Lung Island	4.00	3.92	N.E.	4.0	4.0	N.E.	100	100	N.E.	0.0	16.0	N.E.	12.59	13.06	N.E.	18.9	N.E.	5.4
13	Waglan Island	3.00	2.61	N.E.	3.0	3.0	N.E.	100	100	N.E.	0.0	0.0	N.E.	0.18	1.19	N.E.	20.0	N.E.	11.5
14	Po Toi	3.52	2.76	3.61	4.0	3.0	3.0	100	100	100	0.0	5.9	13.0	2.32	2.19	9.61	18.6	18.6	11.9
15	Shek O	3.90	4.53	4.40	4.0	5.0	4.0	100	100	100	5.0	52.9	35.0	29.65	18.09	21.55	22.5	16.8	4.8
16	Turtle Cove	3.33	3.30	3.74	3.0	3.0	4.0	100	100	100	0.0	10.0	15.8	0.43	3.96	8.05	21.7	19.6	6.4
17	Chung Hum Kok	4.10	4.56	N.E.	4.0	4.5	N.E.	100	100	N.E.	4.8	50.0	N.E.	9.01	10.82	N.E.	25.1	N.E.	4.7
18	Repulse Bay	3.90	3.97	N.E.	4.0	4.0	N.E.	100	100	N.E.	10.5	26.7	N.E.	11.84	11.02	N.E.	24.8	N.E.	3.7
19	Deep Water Bay	4.35	3.45	4.90	4.0	3.0	5.0	100	100	100	29.4	25.0	57.1	7.72	7.10	33.12	15.2	21.9	2.5
20	Aberdeen	5.00	5.05	5.21	5.0	5.0	5.0	100	100	100	83.3	71.4	91.7	64.22	26.05	42.96	22.8	24.9	1.4
21	Sok Kwu Wan	5.36	5.73	5.22	6.0	6.0	5.0	100	100	100	71.4	93.3	94.4	12.07	37.70	38.87	23.8	24.5	0.4
22	Ha Mei Wan	3.65	4.41	N.E.	4.0	4.0	N.E.	100	100	N.E.	0.0	40.7	N.E.	1.89	16.72	N.E.	25.6	N.E.	2.9
23	Mui Wo	4.00	3.05	N.E.	4.0	3.0	N.E.	100	100	N.E.	0.0	5.3	N.E.	3.05	8.76	N.E.	29.0	N.E.	0.8
24	Cheung Sha	4.00	3.79	N.E.	4.0	3.0	N.E.	100	100	N.E.	0.0	26.3	N.E.	1.26	13.73	N.E.	16.8	N.E.	6.2
25	Tai O	3.79	4.17	N.E.	4.0	4.0	N.E.	100	100	N.E.	0.0	39.1	N.E.	3.18	24.80	N.E.	20.0	N.E.	0.2
26	Butterfly Beach	4.11	3.96	4.95	4.0	4.0	5.0	100	100	100	33.3	21.7	60.0	15.56	34.40	46.19	26.8	27.4	1.7
27	Kadoorie Beach	4.87	4.00	4.47	5.0	4.0	5.0	100	100	100	53.3	28.6	52.9	38.43	21.21	43.60	28.9	25.4	0.9
28	Pak Sha Wan	4.46	4.79	N.E.	N.A.	5.0	N.E.	100	100	N.E.	25.0	57.9	N.E.	60.61	46.58	N.E.	24.4	N.E.	0.0
29	Waterfall Bay	4.06	4.87	N.E.	N.A.	5.0	N.E.	100	100	N.E.	6.3	53.3	N.E.	59.80	11.56	N.E.	26.1	N.E.	1.5

Data were collected in 2004–06 (extracted from Leung et al. [14] for sites 1–27 and Qiu et al. [20] for sites 28 and 29), 2010 and 2015 (present study).

N.A.: data not available

N.E.: means not evaluated

Temporally, the females continued displaying 100% imposex from surveys in 2004 to 2015 (Table 1). Site-to-site comparisons showed no differences of VDSI between 2004–06 and 2010 (Wilcoxon signed-rank test: *Z* = 0.330, *p* > 0.05), while VDSI slightly increased during 2010 to 2015 (Paired-samples *t* test: *t* = 2.412, *p* < 0.05). RPSI, however, increased steadily between 2004–06 and 2010 (*t* = 4.350, *p* < 0.001), and between 2010 and 2015 (*t* = 2.429, *p* < 0.05). On the other hand, the percentage of sterile females increased significantly between 2004–2006 and 2010 (*Z* = 3.943, *p* < 0.001), and 8 out of 10 sites continued this increasing trend between 2010 and 2015 (Table 1).

### Tissue OT concentrations and degradation indices

**Fig 2:**
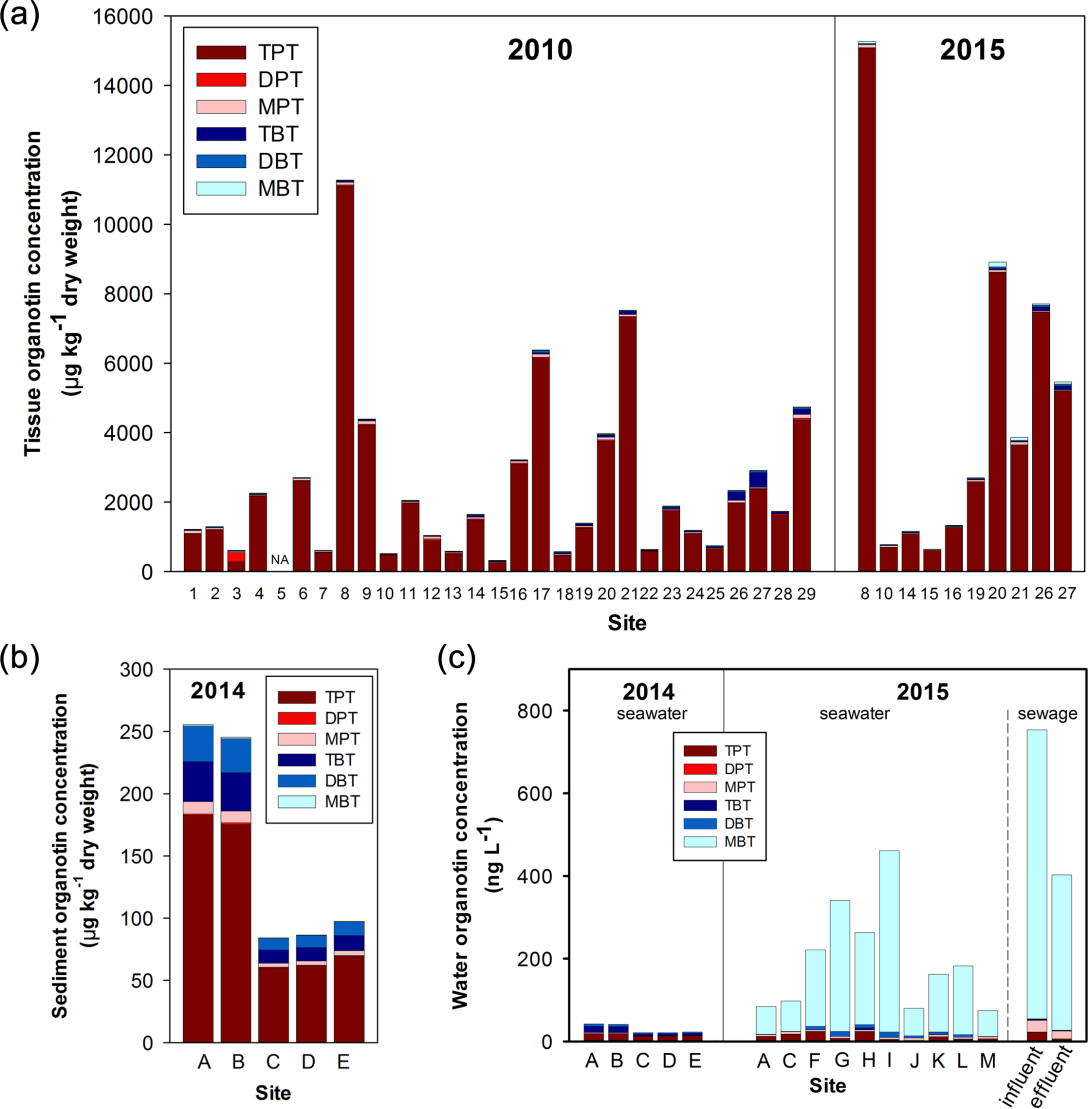
Concentrations of total organotins. These include triphenyltin (TPT), diphenyltin (DPT), monophenyltin (MPT), tributyltin (TBT), diphenyltin (DPT) and monobutyltin (MBT) in (a) tissue samples of *Reishia clavigera* collected in 2010 and 2015 (*n* = 3 or 4); (b) sediment samples collected in 2014 (*n* = 2); and (c) seawater samples collected in 2014 and 2015, plus samples of sewage in/effluent collected in 2015 (*n* = 2 or 3). NA means data not available.

Tissue concentrations of total OTs in *R*. *clavigera* ranged from 318.5–11,278.9 μg kg^-1^ dw in 2010 (equivalent to 24.2–769.0 μg Sn kg^-1^ wet weight (ww)), and from 643.9–15,304.9 μg kg^-1^ dw in 2015 (equivalent to 46.5–1039.4 μg Sn kg^-1^ ww) (Fig 2A). TPT, which ranged from 227.9–11,108.0 μg kg^-1^ dw in 2010 (equivalent to 15.4–751.4 μg Sn kg^-1^ ww) and 612.4 to 15,059.6 μg kg^-1^ dw in 2015 (equivalent to 41.4–1,018.6 μg Sn kg^-1^ ww) respectively, was the predominant residue accounting for 46–99% (2010) and 90–98% (2015) of total OTs. Concentrations of TBT ranged from 5.8 to 422.0 μg kg^-1^ dw in 2010 (equivalent to 0.5–34.5 μg Sn kg^-1^ ww), and from below detection limit to 117.3 μg kg^-1^ dw in 2015 (equivalent to below detection limit–9.6 μg Sn kg^-1^ ww). MBT, DBT, MPT and DPT were also detected (S11 Table for dry weight and S12 Table for wet weight, respectively). Eighteen out of 26 sites had BDI < 1 in 2010, while 9 out of 10 sites in 2015 showed BDI > 1. All sites showed PDI < 1 in 2010 was except in Chek Chau, while they all fell below 1 in 2015 (S13 Table).

Site-to-site comparisons among years showed that tissue TBT concentrations significantly increased during 2004–2006 and 2010 (*t* = 3.637, *p* < 0.001), while it decreased from 2010 to 2015 (*Z* = -2.803, *p* < 0.01). For TPT and total OTs, their concentrations remained similar between 2010 and 2015 (*Z* = 0.866, *p* > 0.05) (S11 and S12 Tables).

### Correlations among OT concentrations, imposex indices and condition index

Tissue concentration of total OTs was positively correlated with TPT concentration (in 2010: *r*_*s*_ = 0.994, *p* < 0.001; S14 Table and in 2015: *r*_*s*_ > 0.999, *p* < 0.001; S15 Table). There was, in general, good agreement among mean VDSI, median VDSI and RPSI although sometimes it was statistically insignificant after Bonferroni corrections. A significant positive relationship was also found between tissue TBT concentration and RPSI (*r*_*s*_ = 0.560, *p* < 0.01) in 2010, and between tissue TPT concentration and RPSI (*r*_*s*_ = 0.855, *p* < 0.01) in 2015 (S14 and S15 Tables).

### Relationships between shipping activities and OT contaminations

In general, imposex status (VDSI, RPSI and percentage of sterile females) and tissue concentrations of OTs decreased with the distance to the nearest facility with high shipping activities as shown in 2010 and 2015, although some combinations showed statistically insignificant results after Bonferroni corrections (S16 Table).

### OT concentrations in seawater and sediment

Total OTs concentrations ranged from 83.7 to 255.5 ng g^-1^ dw in sediment, and 20.5 to 41.9 ng L^-1^ in seawater, respectively measured in 2014. The two sampling stations in Aberdeen (A and B) showed the highest OTs concentrations in seawater and sediment (Fig 2B and 2C). TPT was the most abundant among all OT residues, accounting for 45–63% in seawater and 71–72% in sediment, respectively. TBT accounted for a higher percentage among OTs in seawater (25–37%) than that in sediment (13%).

In 2015, the range of total OTs concentrations was between 74.5 and 753.3 ng L^-1^ in seawater and sewage (Fig 2C; S4 Table). Among all, the influent of the sewage treatment plant showed the highest OTs concentration. MBT was the predominating compound (75–95% of total OTs) in these samples.

### Ecological risk assessment

**Fig 3:**
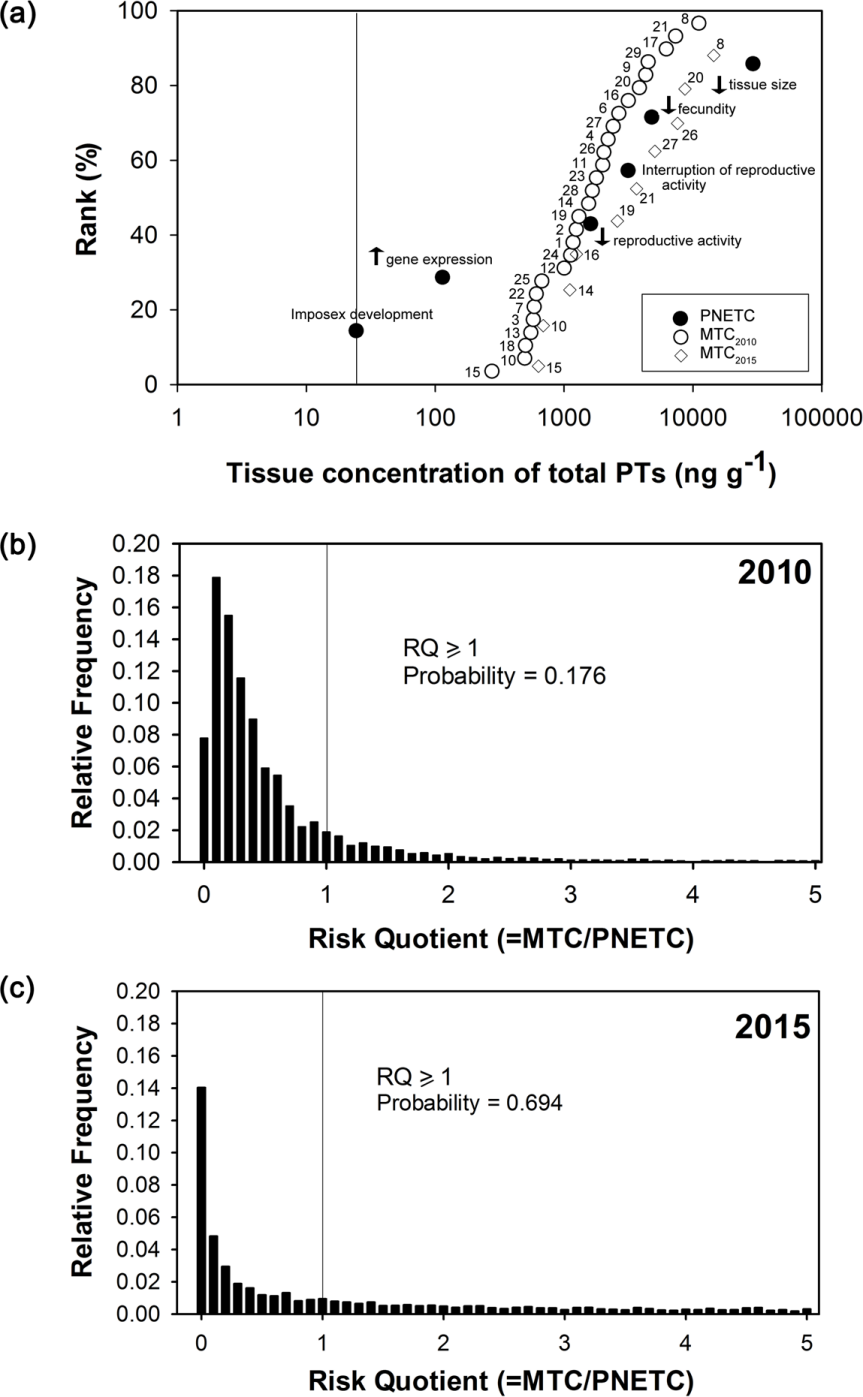
Results of the probabilistic ecological risk assessment. These include (a) species sensitivity distribution to phenyltins (PTs) constructed based on chronic toxicity data from literatures (PNETCs; filled circle) and distribution of tissue concentrations of total PTs in *Reishia clavigera* from this study (MTCs; 2010: open circle; 2015: open diamond); and (b) distribution of the computed risk quotients (RQs) of 2010 and (c) 2015 generated from Monte Carlo simulation. Site numbers (see Table 1) are labelled next to the open circles and diamonds in (a).

Cumulative distributions of six PNETC values were constructed for BTs (S8 Table) and PTs (S9 Table), respectively. In 2010, all 29 sites were seriously impacted by PTs, as shown by the overlap of MTC and PNETC distributions (Fig 3A). This situation continued in 2015, where all 10 sites had MTCs higher than the lowest PNETC. The results of the probabilistic risk assessment suggested that local *R*. *clavigera* collected in 2010 had 17.6% of chance to be at risk from exposure to PTs (i.e., RQ ≥ 1; Fig 3B), while the chance that *R*. *clavigera* being at risk has significantly increased to 69.4% in 2015 (Fig 3C).

**Fig 4:**
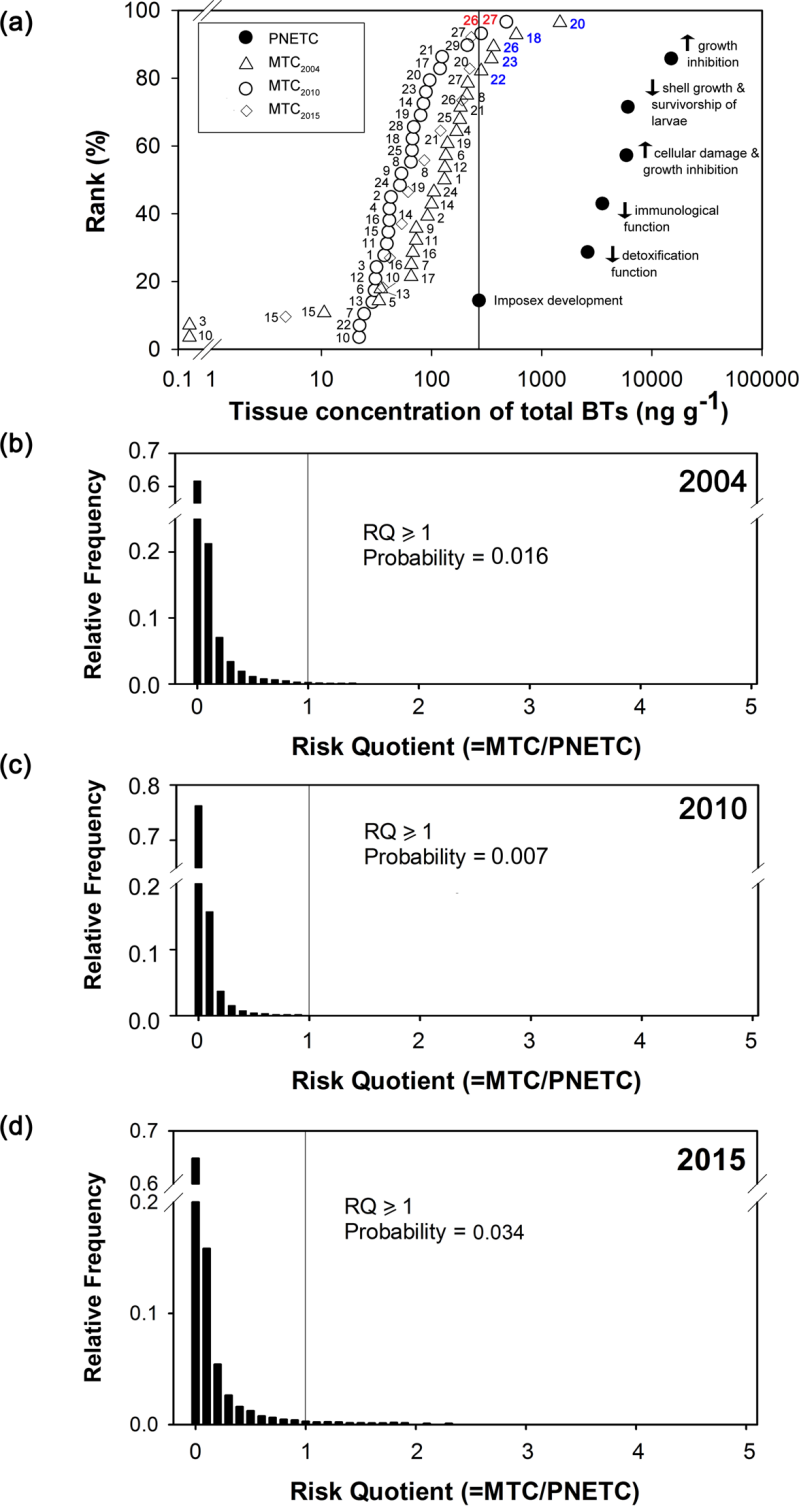
Results of the probabilistic ecological risk assessment. These include (a) species sensitivity distribution to butyltins (BTs) constructed based on chronic toxicity data from literatures (PNETCs; filled circle) and distribution of tissue concentrations of total BTs in *Reishia clavigera* from this study (MTCs; 2010: open circle; 2015: open diamond) and from the study in 2004 (Leung et al. [14]; open triangle); (b) distribution of the computed risk quotients (RQs) of the study in 2004 (modified from Leung et al. [14]), (c) 2010 and (d) 2015 generated from Monte Carlo simulation. Site numbers (see Table 1) are labelled next to the open circles, triangles and diamonds in (a).

Using total BTs instead of TBT, we reconstructed the distribution of tissue concentrations of total BTs and the computed risk quotients for 2004 (data extracted from Leung et al. [12]). The number of sites that was severely impacted by BTs reduced from five (2004) to two (2010), while all 10 sites had MTCs lower than the smallest PNETC in 2015 (Fig 4A). The chance for *R*. *clavigera* being impacted by BTs decreased from 1.6% to 0.7% during 2004 to 2010, but slightly increased to 3.4% in 2015 (Fig 4B–4D).

## Discussion

### Spatial variation of OT contamination

Generally, the eastern waters of Hong Kong, except inner Sai Kung, exhibited lower OT contamination (i.e., low VDSI and RPSI) in *R*. *clavigera*, due to less intense shipping activities and the prevailing ocean current helped mix, dilute and wash away the pollutants. The western waters, however, were more polluted due to (1) intense shipping activities around the container terminals in Kwai Chung, Tsing Yi and Tuen Mun and (2) contaminated freshwater input from the Pearl River Delta (PRD) originated from agricultural and industrial activities. Conventionally, the water, sediment and biota in PRD were heavily contaminated by OTs [26]. For example, TBT concentration in PRD’s estuarine water could reach 38.5 ng L^-1^ which was 4–20 folds higher than the environmental standards in the United States and United Kingdom [27]. We also detected higher concentrations of OTs in seawater samples (over 200 ng L^-1^) from the western waters of Hong Kong (sites F, G, H and I; Fig 2C).

Specifically, areas of fish villages and typhoon shelters with intense shipping activities such as Sok Kwu Wan and Aberdeen, exhibited the highest levels of VDSI in *R*. *clavigera*. Sai Kung Pier, which recorded the highest RPSI and tissue concentrations of TPT and total OTs, is a hotspot for ferries and leisure boats especially during summer. The highest TBT levels in *R*. *clavigera* were observed in Kadoorie Beach and Butterfly Beach, where are close to the typhoon shelter in Tuen Mun. These results were in line with the lowest PDI and BDI found in these areas, indicating recent inputs of TPT and TBT, respectively. The findings were also supported by the highest TBT concentration and the second highest TPT concentration found in seawater in the same location (site H). Moreover, the positive association between OT contamination and proximity to shipping activities was consistent with previous studies [28], and this further supported that marine shipping activities are highly possibly a major source of OT contamination. The input of TPT might reflect the replacement of TPT from TBT as the active antifouling agent in Hong Kong and South China which deserves further investigations on the ingredients of the currently used antifouling paints.

The concentrations of OTs in sediment and in seawater measured in this territorial-wide study, i.e., in sediment (83.7–255.5 ng g^-1^ dw) and in seawater samples of 2014 (20.5–41.9 ng L^-1^) and 2015 (74.5–461.1 ng L^-1^), were much higher than those measured in a parallel study that sampled in four marine protected areas in Hong Kong (46.2–126.4 ng g^-1^ dw in sediment and 0.8–7.8 ng L^-1^ in seawater; Xu et al., unpublished results). Considering the results in 2015, the highest concentrations of butyltins (mainly MBT and DBT) in water were linked to stormwater drainage (site F, G), nullah discharge (site K and I) and sewage (influent and effluent of treatment plant). This could be due to the industrial and domestic releases of such compounds from PVC pipes, antifungal coatings on textiles and wood preservatives [29] especially during the wet summer season when there were increased amounts of rainfall and surface runoff. Similarly, we observed increased concentrations of nonylphenols in a marine reserve in Hong Kong during summer [30]. In contrast, phenyltins were likely associated with shipping activities as sites C, F and H showed the highest concentrations among seawater samples, as well as in the influent of the sewage treatment plant (S4 Table). In Hong Kong, over 80% of population uses seawater for toilet flushing and such saline wastewater enters sewage treatment plants [31]. Thus, it is not surprised to see the sewage influent being contaminated with organotins. Overall, OT contamination is still a widespread problem in Hong Kong which requires more in-depth investigations on the sources of these pollutants.

### Temporal trends of OT contamination

We consistently showed 100% imposex in female *R*. *clavigera* and further noted the increases of imposex indices and the percentage of sterile females in 2010 and 2015, suggesting that OT contamination is persistent. Interestingly, although there was a reduction of TBT concentration in *R*. *clavigera* during 2010 to 2015, the ecological risks of the rock shell populations exposed to BTs increased over the period. This is attributed to the increases in concentrations of MBT and DBT, in which the former compound is the most abundant among all OTs found in the seawater samples. However, in certain less impacted sites such as Shek Mei Tao, Clear Water Bay and Chek Chau, there were elevated tissue concentrations of TBT in 2010 comparing to those in 2004 and the maximum amount of increase was 384.5 μg kg^-1^ dw, or 195 folds. This may be attributed to the proximity of these sites towards the fish culture zones in Po Toi O and Tap Mun. Although TBT was detected at low concentrations in the seawater samples in the present study, it could be due to the fact that TBT is easily degraded into DBT, and further into MBT in seawater as compared to their degradation rates in sediment and biota [30]. Moreover, marine organisms can readily take up the pollutants once they passively diffused from sediment into seawater [32] in addition to the terrestrial sources of butyltins. The lack of regulations to control the use and release of these compounds [33], or the lack of proper enforcement of existing regulations [34] worsens the situation. Not surprisingly, high levels of TBT were still detected in Asia where there is a lack of comprehensive regulations on its use and release [35, 36], or there are antifouling fragments remained in shipyards [37] and illegal uses of TBT in places such as mariculture farms [38].

As TPT is applied as co-toxicant in antifouling systems with TBT [39], these two compounds often coexisted in marine organisms with high concentrations [40], and both have comparative potencies in triggering imposex in *R*. *clavigera* [5, 41]. The present study was the first one quantifying concentrations of various PTs (i.e., MPT, DPT and TPT) in the tissues of *R*. *clavigera* and in coastal water and sediment from Hong Kong. In the past, TPT and its degradation products were seldom measured in any compartment (i.e., water, sediment and biota) of local waters. Nakayama et al. first reported higher TPT concentration (up to 400 ng g^-1^ ww) in the liver of the finless porpoise (*Neophocaena phocaenoides*) collected locally as compared to cetaceans from other Asian locations [42]. Recent studies also documented TPT as the dominating compounds among OTs in several gastropod species including *R*. *clavigera* collected in Shenzhen [43, 44], and in several seafood species sampled in Hong Kong [23] suggesting that the coastal marine environment of South China was severely contaminated with TPT. The present study confirmed the high concentrations of TPT not only in marine organisms but also in seawater and sediment from Hong Kong waters, whereas the concentrations of TBT relatively low.

### Comparison of OT contamination with other regions

We found fluctuations in concentrations of TBT in *R*. *clavigera*. This does not agree with the global decreasing trend [45] which could possibly due to the terrestrial input of TBT and the passive diffusion from sediment. While plentiful information of TBT is available, only limited monitoring studies had incorporated measurements of TPT. Shim et al. recorded TPT concentration in *R*. *clavigera* up to 2,460 ng Sn g^-1^ dw (ca. 7,256 μg TPT kg^-1^ dw) in Korea [40], which was comparable to the highest TPT concentration (i.e., 15,060 μg kg^-1^ dw) measured in the present study. In Japan, however, tissue TPT concentrations in *R*. *clavigera* were relatively low and decreasing [46]. Nonetheless, notable amount of TPT, with comparison of TBT, was detected in sediment from a Japanese fishing port [47]. In agreement with the present study, higher tissue concentrations of TPT, compared to TBT, were also detected in *R*. *clavigera* from Korea [40] and Taiwan [48]. In fishes collected from Bohai Bay, China, TPT concentrations (34.7 ng g^-1^ wet weight) were higher than those of TBT [49], but they were much lower than the concentrations measured in gastropods in the present study. This suggested that contamination of TPT could be a widespread problem in Asia. A more comprehensive study covering a wider range of areas is needed to understand the environmental fate and contemporary sources of OTs especially TPT in Asian coasts.

### Implications for management action

Being an associate member of IMO which has yet to ratify the AFS Convention [50], Hong Kong demonstrated to other countries or areas of similar status, such as Thailand, Vietnam and Philippines, that only internationally-bound regulatory measures are not sufficient to control chemical contamination, such as OTs, in regional scale. Based on the experience from the US, Europe and Japan, local legislations in controlling the use of OTs as antifouling paints, pesticides, fungicides and other industrial uses should be more promising and effective in reducing OT contamination [51]. The Legislative Council of Hong Kong passed the Merchant Shipping (Control of Harmful Anti-Fouling Systems on Ships) Regulation (under Cap. 413) on 3 June 2015, and the government is now requesting the Central People's Government of China to notify the IMO the extension of the Convention to Hong Kong [52]. The present study, therefore, call for urgent implementation of the Convention and appropriate management actions to remediate the pollution and impacts of these compounds in the marine environment of this region.

## Conclusions

Although OTs, as antifouling agents, were banned globally, they have been contaminating the coastal marine environment of Hong Kong over the past two decades and causing ecological risk to local rock shell populations. This study demonstrated that the implementation of the international regulation alone may not be effective enough in controlling widespread environmental problems, local and regional management actions are urgently needed to remediate the pollution of these compounds from various sources.

## Supporting Information

S1 Table Geographical and morphological information of *Reishia clavigera* collected in Hong Kong in 2010. (DOCX)

S2 Table Geographical and morphological information of *Reishia clavigera* collected in Hong Kong in 2015. (DOCX)

S3 Table Method of evaluating Vas Deferens Sequence Index (VDSI) and Relative Penis Size Index (RPSI). (DOCX)

S4 Table Concentrations of organotins in water. (DOCX)

S5 Table Chemical analyses (sample collection, extraction and clean-up) of organotin in samples of rock shell and sediment, and in water. (DOCX)

S6 Table Analytical parameters of gas chromatography-mass spectrometer. (DOCX)

S7 Table Average recoveries of spiked organotin standards into clean mussel samples. (DOCX)

S8 Table Method of evaluating risk quotient. (DOCX)

S9 Table Chronic and sub-chronic toxicity values of body concentration of tributyltin for molluscs. (DOCX)

S10 Table Chronic and sub-chronic toxicity values of body concentration of triphenyltin for molluscs. (DOCX)

S11 Table Tissue concentrations (in μg kg^-1^ dry weight) of six organotins (OTs): monobutyltin (MBT), dibutyltin (DBT), tributyltin (TBT), monophenyltin (MPT), diphenyltin (DPT) and triphenyltin (TPT) in *Reishia clavigera* collected in 2004–06, 2010 and 2015 from Hong Kong. (DOCX)

S12 Table Tissue concentrations (in μg Sn kg^-1^ wet weight) of six organotins (OTs): monobutyltin (MBT), dibutyltin (DBT), tributyltin (TBT), monophenyltin (MPT), diphenyltin (DPT) and triphenyltin (TPT) in *Reishia clavigera* collected in 2004–06, 2010 and 2015 from Hong Kong. (DOCX)

S13 Table Degradation indices (Butyltin Degradation Index, BDI and Phenyltin Degradation Index, PDI) of butyltin and phenyltin compounds in the tissues of *Reishia clavigera*. (DOCX)

S14 Table Results of Spearman’s rank correlation analyses among tissue concentrations of total organotins (total OTs), triphenyltin (TPT), diphenyltin (DPT), tributyltin (TBT), Relative Penis Size Index (RPSI), median Vas Deferens Sequence Index (VDSI), mean VDSI and condition index (CI) in *Reishia clavigera* collected in 2010. (DOCX)

S15 Table Results of Spearman’s rank correlation analyses among tissue concentrations of total organotins (total OTs), triphenyltin (TPT), diphenyltin (DPT), tributyltin (TBT), Relative Penis Size Index (RPSI), median Vas Deferens Sequence Index (VDSI), mean VDSI and condition index (CI) in *Reishia clavigera* collected in 2015. (DOCX)

S16 Table Spearman’s rank correlation analyses between the distance to major shipping activities and imposex status (including Vas Deferens Sequence Index (VDSI) and Relative Penis Size Index (RPSI)), condition index and tissue concentrations of organotins. (DOCX)

S17 Table List of references of Supporting Information (S1–S16 Tables). (DOCX)
