# Supplementary material for: Long-Term Spatio-Temporal Trends of Organotin Contaminations in the Marine Environment of Hong Kong
Source: PLoS One. 2016 May 13;11(5):e0155632. doi: 10.1371/journal.pone.0155632 (PMC4866715; doi:10.1371/journal.pone.0155632)
Supplement: S1 Table — (DOCX) [file pone.0155632.s001.docx]

**S1 Table.** **Geographical and morphological information of *Reishia clavigera* collected in Hong Kong in 2010.** N.A. means data not available.

| **Site** | | **GPS coordinates** | | ***N*** | **(Male:Female)** | **Sampling date** |
| --- | --- | --- | --- | --- | --- | --- |
|  |  | **Latitude** | **Longitude** |  |  |  |
| 1 | Kat O | 22°33'03.8"N | 114°17'50.1"E | 40 | (15:25) | 2010.8.9 |
| 2 | Pak Sha Chau | 22°32'31.0"N | 114°19'52.1"E | 40 | (26:14) | 2010.8.9 |
| 3 | Chek Chau | 22°30'14.5"N | 114°21'44.6"E | 40 | (19:21) | 2010.8.9 |
| 4 | Wu Kwai Sha | 22°26'04.9"N | 114°14'49.3"E | 40 | (22:18) | 2010.7.29 |
| 5 | Heng On | 22°24'26.4"N | 114°13'09.1"E | 0 | N.A. | 2010.7.29 |
| 6 | Wong Mau Chau | 22°26'54.4"N | 114°23'42.7"E | 17 | (6:11) | 2010.8.9 |
| 7 | Kong Tau Pai | 22°20'21.1"N | 114°22'28.3"E | 40 | (14:26) | 2010.9.8 |
| 8 | Sai Kung Pier | 22°23'01.5"N | 114°16'38.0"E | 40 | (22:18) | 2010.7.29 |
| 9 | UST | 22°20'18.7"N | 114°16'01.1"E | 36 | (16:20) | 2010.8.6 |
| 10 | Clear Water Bay | 22°17'24.9"N | 114°17'25.2"E | 40 | (19:21) | 2010.8.6 |
| 11 | Shek Mei Tao | 22°16'40.7"N | 114°17'51.9"E | 40 | (16:24) | 2010.8.6 |
| 12 | Tung Lung Island | 22°15'29.8"N | 114°17'34.3"E | 40 | (15:25) | 2010.9.6 |
| 13 | Waglan Island | 22°10'58.6"N | 114°18'10.0"E | 40 | (7:33) | 2010.9.6 |
| 14 | Po Toi | 22°09'47.6"N | 114°15'12.4"E | 40 | (23:17) | 2010.9.6 |
| 15 | Shek O | 22°13'43.5"N | 114°15'12.7"E | 40 | (23:17) | 2010.6.14 |
| 16 | Turtle Cove | 22°13'57.0"N | 114°13'26.7"E | 40 | (20:20) | 2010.6.14 |
| 17 | Chung Hum Kok | 22°13'00.5"N | 114°12'05.9"E | 40 | (22:18) | 2010.6.15 |
| 18 | Repulse Bay | 22°14'04.4"N | 114°11'52.9"E | 40 | (10:30) | 2010.6.15 |
| 19 | Deep Water Bay | 22°14'31.5"N | 114°11'14.2"E | 40 | (20:20) | 2010.6.15 |
| 20 | Aberdeen | 22°14'11.5"N | 114°09'14.7"E | 40 | (19:21) | 2010.7.26 |
| 21 | Sok Kwu Wan | 22°12'13.2"N | 114°07'44.5"E | 35 | (20:15) | 2010.6.24 |
| 22 | Ha Mei Wan | 22°12'13.1"N | 114°07'18.7"E | 40 | (13:27) | 2010.6.24 |
| 23 | Mui Wo | 22°16'18.3"N | 114°00'13.7"E | 40 | (21:19) | 2010.6.11 |
| 24 | Cheung Sha | 22°13'54.2"N | 113°57'26.3"E | 40 | (21:19) | 2010.6.11 |
| 25 | Tai O | 22°14'45.6"N | 113°51'37.1"E | 40 | (17:23) | 2010.6.11 |
| 26 | Butterfly Beach | 22°22'22.4"N | 113°57'36.4"E | 40 | (17:23) | 2010.5.27 |
| 27 | Kadoorie Beach | 22°22'32.9"N | 113°58'56.4"E | 36 | (15:21) | 2010.5.27 |
| 28 | Pak Sha Wan | 22°21'45.7"N | 114°15'35.5"E | 40 | (21:19) | 2010.7.29 |
| 29 | Waterfall Bay | 22°15'08.1"N | 114°07'58.8"E | 40 | (25:15) | 2010.7.26 |

**S1 Table**. (continued)

| **Site** | | **Male** | | |  |  |  | |  | |  |  | |  | |  |  | **Female** | | |  |  |  | |  |  |  | |  |  |
| --- | --- | --- | --- | --- | --- | --- | --- | --- | --- | --- | --- | --- | --- | --- | --- | --- | --- | --- | --- | --- | --- | --- | --- | --- | --- | --- | --- | --- | --- | --- |
|  |  | **Shell length (mm)** | | | | | **Tissue weight (g)** | | | | | **Penis length (mm)** | | | | |  | **Shell length (mm)** | | | | | **Tissue weight (g)** | | | | **Penis length (mm)** | | | |
|  |  | **Mean** | ± | **SD** | | | **Mean** | ± | | **SD** | | **Mean** | ± | | **SD** | |  | **Mean** | ± | **SD** | | | **Mean** | ± | **SD** | | **Mean** | | ± | **SD** |
| 1 | Kat O | 29.2 | ± | 2.8 | | | 0.868 | ± | | 0.308 | | 13.03 | ± | | 2.13 | |  | 30.0 | ± | 2.8 | | | 0.923 | ± | 0.238 | | 5.91 | | ± | 1.76 |
| 2 | Pak Sha Chau | 24.9 | ± | 2.3 | | | 0.620 | ± | | 0.177 | | 12.41 | ± | | 2.59 | |  | 26.0 | ± | 3.1 | | | 0.714 | ± | 0.211 | | 5.38 | | ± | 1.07 |
| 3 | Chek Chau | 28.6 | ± | 4.0 | | | 0.902 | ± | | 0.403 | | 14.27 | ± | | 4.02 | |  | 29.0 | ± | 4.3 | | | 0.941 | ± | 0.434 | | 5.16 | | ± | 1.74 |
| 4 | Wu Kwai Sha | 27.8 | ± | 2.3 | | | 0.903 | ± | | 0.262 | | 13.32 | ± | | 4.41 | |  | 29.6 | ± | 2.1 | | | 0.966 | ± | 0.211 | | 5.21 | | ± | 2.31 |
| 5 | Heng On | N.A. | | | | | | | | | | | | | | | | | | | | | | | | | | | | |
| 6 | Wong Mau Chau | 28.2 | ± | 5.1 | | | 0.669 | ± | | 0.328 | | 11.83 | ± | | 6.55 | |  | 30.1 | ± | 3.6 | | | 1.005 | ± | 0.364 | | | 4.1 | ± | 1.61 |
| 7 | Kong Tau Pai | 22.9 | ± | 1.9 | | | 0.460 | ± | | 0.082 | | 10.89 | ± | | 4.62 | |  | 24.7 | ± | 2.2 | | | 0.622 | ± | 0.188 | | | 4.35 | ± | 1.34 |
| 8 | Sai Kung Pier | 28.9 | ± | 3.1 | | | 0.723 | ± | | 0.287 | | 10.83 | ± | | 2.98 | |  | 28.6 | ± | 2.7 | | | 0.744 | ± | 0.172 | | | 10.63 | ± | 2.81 |
| 9 | UST | 25.4 | ± | 3.4 | | | 0.634 | ± | | 0.279 | | 10.90 | ± | | 2.24 | |  | 26.7 | ± | 3.9 | | | 0.692 | ± | 0.239 | | | 8.57 | ± | 1.25 |
| 10 | Clear Water Bay | 25.2 | ± | 2.7 | | | 0.598 | ± | | 0.217 | | 13.25 | ± | | 4.78 | |  | 25.4 | ± | 2.8 | | | 0.660 | ± | 0.216 | | | 3.41 | ± | 2.38 |
| 11 | Shek Mei Tao | 29.4 | ± | 2.9 | | | 0.937 | ± | | 0.283 | | 13.19 | ± | | 3.48 | |  | 28.9 | ± | 3.8 | | | 0.879 | ± | 0.430 | | | 4.24 | ± | 1.86 |
| 12 | Tung Lung Island | 29.8 | ± | 2.9 | | | 0.876 | ± | | 0.247 | | 14.93 | ± | | 4.69 | |  | 28.8 | ± | 2.3 | | | 0.806 | ± | 0.227 | | | 7.58 | ± | 2.51 |
| 13 | Waglan Island | 27.4 | ± | 4.4 | | | 0.847 | ± | | 0.300 | | 16.12 | ± | | 4.72 | |  | 28.0 | ± | 3.5 | | | 0.899 | ± | 0.350 | | | 3.68 | ± | 1.34 |
| 14 | Po Toi | 27.9 | ± | 2.2 | | | 0.764 | ± | | 0.159 | | 14.51 | ± | | 2.60 | |  | 27.5 | ± | 2.9 | | | 0.748 | ± | 0.203 | | | 4.06 | ± | 1.68 |
| 15 | Shek O | 25.8 | ± | 3.5 | | | 0.788 | ± | | 0.278 | | 14.66 | ± | | 2.66 | |  | 28.0 | ± | 3.4 | | | 0.982 | ± | 0.295 | | | 8.29 | ± | 2.83 |
| 16 | Turtle Cove | 27.5 | ± | 2.8 | | | 0.785 | ± | | 0.259 | | 12.80 | ± | | 3.44 | |  | 26.9 | ± | 3.7 | | | 0.778 | ± | 0.384 | | | 4.36 | ± | 1.37 |
| 17 | Chung Hum Kok | 25.4 | ± | 2.8 | | | 0.728 | ± | | 0.245 | | 14.06 | ± | | 2.45 | |  | 25.1 | ± | 2.3 | | | 0.665 | ± | 0.195 | | | 6.7 | ± | 1.79 |
| 18 | Repulse Bay | 24.2 | ± | 1.8 | | | 0.618 | ± | | 0.141 | | 12.77 | ± | | 2.11 | |  | 25.7 | ± | 3.9 | | | 0.779 | ± | 0.349 | | | 6.12 | ± | 1.64 |
| 19 | Deep Water Bay | 29.2 | ± | 4.3 | | | 0.706 | ± | | 0.293 | | 11.42 | ± | | 3.26 | |  | 29.5 | ± | 4.0 | | | 0.678 | ± | 0.293 | | | 4.73 | ± | 2.64 |
| 20 | Aberdeen | 26.9 | ± | 4.0 | | | 0.746 | ± | | 0.408 | | 12.20 | ± | | 2.37 | |  | 28.3 | ± | 3.5 | | | 0.931 | ± | 0.376 | | | 7.79 | ± | 1.72 |
| 21 | Sok Kwu Wan | 32.6 | ± | 2.9 | | | 1.288 | ± | | 0.340 | | 15.81 | ± | | 3.02 | |  | 35.4 | ± | 4.4 | | | 1.570 | ± | 0.454 | | | 11.42 | ± | 2.47 |
| 22 | Ha Mei Wan | 26.4 | ± | 3.4 | | | 0.821 | ± | | 0.318 | | 14.09 | ± | | 2.57 | |  | 28.6 | ± | 4.4 | | | 1.072 | ± | 0.470 | | | 7.76 | ± | 1.67 |
| 23 | Mui Wo | 24.8 | ± | 2.5 | | | 0.770 | ± | | 0.230 | | 11.78 | ± | | 3.03 | |  | 25.6 | ± | 2.4 | | | 0.860 | ± | 0.231 | | | 5.23 | ± | 2.32 |
| 24 | Cheung Sha | 31.4 | ± | 6.1 | | | 1.057 | ± | | 0.696 | | 13.63 | ± | | 6.66 | |  | 35.7 | ± | 9.9 | | | 1.745 | ± | 1.500 | | | 7.03 | ± | 3.98 |
| 25 | Tai O | 31.1 | ± | 2.9 | | | 1.004 | ± | | 0.291 | | 14.07 | ± | | 2.51 | |  | 31.6 | ± | 3.6 | | | 1.056 | ± | 0.344 | | | 8.84 | ± | 2.31 |
| 26 | Butterfly Beach | 24.0 | ± | 2.6 | | | 0.628 | ± | | 0.244 | | 11.48 | ± | | 4.70 | |  | 25.7 | ± | 2.7 | | | 0.799 | ± | 0.236 | | | 8.04 | ± | 1.97 |
| 27 | Kadoorie Beach | 26.8 | ± | 3.4 | | | 0.954 | ± | | 0.500 | | 15.30 | ± | | 2.10 | |  | 28.0 | ± | 2.6 | | | 1.197 | ± | 0.386 | | | 9.1 | ± | 3.70 |
| 28 | Pak Sha Wan | 32.0 | ± | 3.3 | | | 1.273 | ± | | 0.444 | | 11.25 | ± | | 3.19 | |  | 32.0 | ± | 2.5 | | | 1.251 | ± | 0.343 | | | 8.72 | ± | 2.78 |
| 29 | Waterfall Bay | 28.6 | ± | 5.3 | | | 1.112 | ± | | 0.631 | | 14.95 | ± | | 2.87 | |  | 27.5 | ± | 3.4 | | | 0.352 | ± | 0.352 | | | 7.29 | ± | 2.06 |
