# Supplementary material for: Long-Term Spatio-Temporal Trends of Organotin Contaminations in the Marine Environment of Hong Kong
Source: PLoS One. 2016 May 13;11(5):e0155632. doi: 10.1371/journal.pone.0155632 (PMC4866715; doi:10.1371/journal.pone.0155632)
Supplement: S2 Table — (DOCX) [file pone.0155632.s002.docx]

**S2 Table.** Geographical and morphological information of *Reishia clavigera* collected in Hong Kong in 2015.

| **Site** | | **GPS coordinates** | | ***N*** | **(Male:Female)** | **Sampling date** |
| --- | --- | --- | --- | --- | --- | --- |
|  |  | **Latitude** | **Longitude** |  |  |  |
| 8 | Sai Kung Pier | 22°23'01.5"N | 114°16'38.0"E | 25 | (16:9) | 2015.7.29 |
| 10 | Clear Water Bay | 22°17'24.9"N | 114°17'25.2"E | 40 | (23:17) | 2015.7.29 |
| 14 | Po Toi | 22°09'47.6"N | 114°15'12.4"E | 40 | (17:23) | 2015.7.28 |
| 15 | Shek O | 22°13'43.5"N | 114°15'12.7"E | 40 | (20:20) | 2015.7.31 |
| 16 | Turtle Cove | 22°13'57.0"N | 114°13'26.7"E | 40 | (21:19) | 2015.7.31 |
| 19 | Deep Water Bay | 22°14'31.5"N | 114°11'14.2"E | 39 | (18:21) | 2015.7.31 |
| 20 | Aberdeen | 22°14'11.5"N | 114°09'14.7"E | 40 | (16:24) | 2015.7.31 |
| 21 | Sok Kwu Wan | 22°12'13.2"N | 114°07'44.5"E | 40 | (22:18) | 2015.8.1 |
| 26 | Butterfly Beach | 22°22'22.4"N | 113°57'36.4"E | 40 | (20:20) | 2015.7.30 |
| 27 | Kadoorie Beach | 22°22'32.9"N | 113°58'56.4"E | 40 | (23:17) | 2015.7.30 |

**S2 Table.** (continued)

| **Site** | | **Male** | | |  |  |  | |  | |  |  | |  | |  |  | **Female** | | |  |  |  | |  |  |  | |  |  |
| --- | --- | --- | --- | --- | --- | --- | --- | --- | --- | --- | --- | --- | --- | --- | --- | --- | --- | --- | --- | --- | --- | --- | --- | --- | --- | --- | --- | --- | --- | --- |
|  |  | **Shell length (mm)** | | | | | **Tissue weight (g)** | | | | | **Penis length (mm)** | | | | |  | **Shell length (mm)** | | | | | **Tissue weight (g)** | | | | **Penis length (mm)** | | | |
|  |  | **Mean** | ± | **SD** | | | **Mean** | ± | | **SD** | | **Mean** | ± | | **SD** | |  | **Mean** | ± | **SD** | | | **Mean** | ± | **SD** | | **Mean** | | ± | **SD** |
| 8 | Sai Kung Pier | 29.7 | ± | 4.98 | | | 1.006 | ± | | 0.509 | | 11.31 | ± | | 3.18 | |  | 29.0 | ± | 2.46 | | | 0.897 | ± | 0.303 | | | 8.86 | ± | 3.26 |
| 10 | Clear Water Bay | 25.3 | ± | 3.67 | | | 0.709 | ± | | 0.313 | | 13.01 | ± | | 2.65 | |  | 24.0 | ± | 3.84 | | | 0.737 | ± | 0.371 | | | 8.87 | ± | 2.26 |
| 14 | Po Toi | 26.1 | ± | 2.34 | | | 0.655 | ± | | 0.158 | | 13.11 | ± | | 1.62 | |  | 25.7 | ± | 3.71 | | | 0.656 | ± | 0.320 | | | 6.01 | ± | 1.50 |
| 15 | Shek O | 28.5 | ± | 5.04 | | | 0.820 | ± | | 0.454 | | 13.61 | ± | | 2.25 | |  | 27.6 | ± | 3.35 | | | 0.814 | ± | 0.393 | | | 8.16 | ± | 2.37 |
| 16 | Turtle Cove | 24.9 | ± | 2.90 | | | 0.568 | ± | | 0.186 | | 13.27 | ± | | 2.29 | |  | 25.6 | ± | 3.38 | | | 0.596 | ± | 0.227 | | | 5.73 | ± | 1.24 |
| 19 | Deep Water Bay | 25.4 | ± | 1.92 | | | 0.616 | ± | | 0.145 | | 13.08 | ± | | 1.94 | |  | 27.0 | ± | 2.58 | | | 0.845 | ± | 0.242 | | | 9.05 | ± | 1.93 |
| 20 | Aberdeen | 28.6 | ± | 3.51 | | | 0.993 | ± | | 0.431 | | 11.88 | ± | | 1.88 | |  | 27.3 | ± | 2.82 | | | 0.940 | ± | 0.293 | | | 8.96 | ± | 1.42 |
| 21 | Sok Kwu Wan | 29.0 | ± | 2.23 | | | 0.912 | ± | | 0.252 | | 14.36 | ± | | 2.52 | |  | 30.6 | ± | 3.46 | | | 1.097 | ± | 0.371 | | | 10.48 | ± | 2.36 |
| 26 | Butterfly Beach | 22.8 | ± | 1.63 | | | 0.548 | ± | | 0.123 | | 10.59 | ± | | 1.32 | |  | 23.6 | ± | 2.40 | | | 0.636 | ± | 0.227 | | | 8.14 | ± | 0.86 |
| 27 | Kadoorie Beach | 25.6 | ± | 2.01 | | | 0.751 | ± | | 0.219 | | 11.75 | ± | | 1.85 | |  | 25.0 | ± | 2.34 | | | 0.761 | ± | 0.254 | | | 8.91 | ± | 2.05 |
