# Supplementary material for: Long-Term Spatio-Temporal Trends of Organotin Contaminations in the Marine Environment of Hong Kong
Source: PLoS One. 2016 May 13;11(5):e0155632. doi: 10.1371/journal.pone.0155632 (PMC4866715; doi:10.1371/journal.pone.0155632)
Supplement: S3 Table — (DOCX) [file pone.0155632.s003.docx]

**S3 Table.**  **Method of evaluating Vas Deferens Sequence Index (VDSI) and Relative Penis Size Index (RPSI).**

| VDSI | VDSI measures the progressive imposex development in gastropods by seven stages where stage 0 indicates no imposex development and stage 6 represents the retention of aborted eggs in capsule gland. Stages 5 and 6 indicate the infertility of females due to the blockage of the oviduct opening (Gibbs et al., 1987; Cheung et al., 2010). |
| --- | --- |
| RPSI | RPSI is the ratio between the mean bulk of female penis and that of male penis (Gibbs et al., 1991), which equals (Mean length of female penis)^3^ × 100 / (Mean length of male penis)^3^. |
