# Supplementary material for: Long-Term Spatio-Temporal Trends of Organotin Contaminations in the Marine Environment of Hong Kong
Source: PLoS One. 2016 May 13;11(5):e0155632. doi: 10.1371/journal.pone.0155632 (PMC4866715; doi:10.1371/journal.pone.0155632)
Supplement: S4 Table — (DOCX) [file pone.0155632.s004.docx]

**S4 Table. Concentrations of organotins (OTs, in unit of ng L^-1^) in water.** These include mono-, di- and tri-butyltin (MBT, DBT and TBT respectively), and mono-, di-, and tri-phenyltin (MPT, DPT and TPT respectively) collected in Hong Kong waters during August 2015. Site locations refer to Fig 1. Influent and effluent were from Stanley Wastewater Treatment Works. B.D.L. means below detection limit.

| **Site** | | **GPS coordinates** | | **MBT** | **DBT** | **TBT** | **Total BTs** | **MPT** | **DPT** | **TPT** | **Total PTs** | **Total OTs** |
| --- | --- | --- | --- | --- | --- | --- | --- | --- | --- | --- | --- | --- |
|  | | **Latitude** | **Longitude** |  |  |  |  |  |  |  |  |  |
|  | A | 22°14'52.0"N | 114°08'56.1"E | 66.9 | B.D.L. | B.D.L. | 67.9 | 4.1 | B.D.L. | 11.7 | 16.3 | 84.2 |
|  | B | 22°14'44.7"N | 114°09'02.7"E | Not quantified | | | | | | | | |
|  | C | 22°22'51.6"N | 114°16'29.2"E | 73.5 | B.D.L. | B.D.L. | 74.5 | 5.3 | B.D.L. | 17.5 | 23.2 | 97.7 |
|  | D | 22°23'01.0"N | 114°16'35.6"E | Not quantified | | | | | | | | |
|  | E | 22°16'17.2"N | 114°14'41.7"E | Not quantified | | | | | | | | |
|  | F | 22°19'42.4"N | 114°08'43.4"E | 185.0 | 8.0 | B.D.L. | 193.2 | 3.9 | B.D.L. | 23.8 | 28.2 | 221.3 |
|  | G | 22°22'04.8"N | 114°05'41.2"E | 316.6 | 12.1 | B.D.L. | 328.9 | 4.1 | B.D.L. | 7.4 | 12.0 | 340.9 |
|  | H | 22°22'48.1"N | 113°58'39.1"E | 222.2 | 7.8 | 5.3 | 235.3 | 3.8 | B.D.L. | 23.5 | 27.8 | 263.0 |
|  | I | 22°28'09.4"N | 114°01'48.5"E | 438.3 | 12.9 | B.D.L. | 451.4 | 4.8 | B.D.L. | 4.5 | 9.7 | 461.1 |
|  | J | 22°18'35.6"N | 114°18'06.1"E | 66.4 | 5.2 | B.D.L. | 71.8 | 5.4 | B.D.L. | 2.3 | 8.2 | 80.0 |
|  | K | 22°18'25.7"N | 114°13'17.9"E | 139.8 | 6.4 | B.D.L. | 146.3 | 4.5 | B.D.L. | 11.1 | 16.0 | 162.4 |
|  | L | 22°13'20.8"N | 114°12'53.0"E | 166.1 | 6.2 | B.D.L. | 172.5 | 4.2 | B.D.L. | 5.6 | 10.2 | 182.7 |
|  | M | 22°15'34.1"N | 114°07'43.8"E | 62.8 | B.D.L. | B.D.L. | 63.8 | 4.2 | B.D.L. | 6.2 | 10.8 | 74.5 |
|  | Influent | 22°12'41.8"N | 114°13'01.1"E | 698.5 | B.D.L. | 3.1 | 702.3 | 28.2 | B.D.L. | 22.3 | 51.0 | 753.3 |
|  | Effluent | 22°12'34.9"N | 114°13'07.2"E | 375.3 | B.D.L. | 1.6 | 377.7 | 18.5 | B.D.L. | 5.8 | 24.8 | 402.4 |
