# Supplementary material for: Long-Term Spatio-Temporal Trends of Organotin Contaminations in the Marine Environment of Hong Kong
Source: PLoS One. 2016 May 13;11(5):e0155632. doi: 10.1371/journal.pone.0155632 (PMC4866715; doi:10.1371/journal.pone.0155632)
Supplement: S5 Table — (DOCX) [file pone.0155632.s005.docx]

**S5 Table. Chemical analyses (sample collection, extraction and clean-up) of organotin in samples of rock shell and sediment (Guðmundsdóttir et al., 2011), and in water (with modifications from Okoro et al., 2012).**

| Rock shell and sediment | Soft-body tissues of rock shells and sediment were freeze-dried for 48 h (VirTis #6KBTES-55 freeze dryer, Gardiner, NY, USA). They were stored in a -20 °C freezer before homogenization with a blender. About 0.5 g of the biota or sediment sample was added to 10 mL of tetramethylammonium hydroxide for digestion. The mixture was shaken at 250 rpm in an orbital shaker for 1.5 h at 50 °C to enhance dissolution. Sodium acetate-acetic acid buffer (pH = 4.96) was added before derivation with sodium tetraethylborate and extraction with hexane. The samples were shaken for 20 min, then centrifuged at 5000 rpm for 15 min at < 4 °C. The organic phase was extracted twice by hexane (5 mL x 2 = 10 mL). The organic phases were combined and reduced to 1 mL, followed by an alumina clean-up. A glass column was packed with ca. 1 g of powdered aluminium oxide (Sigma-Aldrich, St. Louis, MO, USA), and topped with 1 cm^3^ of sodium sulphite (Sigma-Aldrich, St. Louis, MO, USA). Sample extract was loaded into the column, and eluted with hexane until 12 mL of eluate was obtained. The eluate was concentrated to 0.1 mL prior to gas chromatographic analysis. |
| --- | --- |
| Water | One litre of each of the seawater samples were taken from 0.5 m below the water surface and stored in amber glass bottles. About 50 mL of methanol was added to each bottle and the pH was adjusted to 2 using 1 M HCl. And then 1 L of water samples was filtered through glass fibre filters (Whatman GF/F, 0.7 μm, UK). After addition of the internal standards (100 ng), the collected water samples were extracted using Waters Oasis HLB cartridges (6 mL, 500 mg sorbent), which were pre-conditioned with 5 mL toluene, 5 mL methanol and 5 mL H_2_O in sequence. The water samples were introduced to the cartridges at a flow rate of 8-10 mL min^-1^. The sample bottle was rinsed twice with two aliquots of 50 mL of 5% (v/v) methanol in ultrapure water, which passed through the cartridge. After passing through the air for at least 1 h, the target compounds were eluted from the cartridges with 10 mL of toluene under gravity. After concentrating to 1 mL under a gentle nitrogen stream, 1 mL of acetate buffer (82 g L^-1^ sodium acetate in water, adjusted to pH 4.5 with acetic acid), and 50 μL of a derivatizing reagent (2 g NaBEt_4_ in 10 mL methanol) were added. The sample mixture was shaken and allowed to react for 30 min. After the addition of 5 mL water, the derivatized compounds were extracted in 1 mL hexane. The mixture was centrifuged for 10 s and the two phases were allowed to separate. The clear upper layer was extracted, and reduced to 0.1 mL before putting into an amber vial for analysis. |
