# Supplementary material for: Long-Term Spatio-Temporal Trends of Organotin Contaminations in the Marine Environment of Hong Kong
Source: PLoS One. 2016 May 13;11(5):e0155632. doi: 10.1371/journal.pone.0155632 (PMC4866715; doi:10.1371/journal.pone.0155632)
Supplement: S6 Table — (DOCX) [file pone.0155632.s006.docx]

**S6 Table. Analytical parameters of gas chromatography-mass spectrometer.**

| **Temperatures** | Source: 230 °C  Quadrupoles: 106 °C  Interface: 250 °C |
| --- | --- |
| **Carrier gas** | Front detector: 60.0 mL / min N_2_ at 250 °C  Back detector: 30.0 mL / min He at 250 °C |
| **Injected sample** | 2 µL |
| **Split** | Off |
| **Oven programme** | 80 °C held for 1 min, increased at 10 °C / min to 270 °C, increased at 10 °C / min to 280 °C, maintained at 280 ^o^C for 5 min |
| **Ion source** | EI (- 70 eV) |
| **Acquisition mode** | FULL SCAN (total mass spectrum acquisition) and SIM (selected ion monitoring) |
| **Ions monitored** | 177.0, 179.0, 197.0, 205.0, 207.0, 235.0, 247.0, 249.0, 253.0, 255.0, 263.0, 291.0, 301.0, 303.0, 347.0, 349.0, 351.0 |
| **SIM width** | 0.7 amu |
