# Supplementary material for: Long-Term Spatio-Temporal Trends of Organotin Contaminations in the Marine Environment of Hong Kong
Source: PLoS One. 2016 May 13;11(5):e0155632. doi: 10.1371/journal.pone.0155632 (PMC4866715; doi:10.1371/journal.pone.0155632)
Supplement: S7 Table — (DOCX) [file pone.0155632.s007.docx]

**S7 Table.** **Average recoveries (*n* = 3) of spiked organotin standards (100 ng) into clean mussel samples.** Organotin standards include monobutyltin (MBT), dibutyltin (DBT), tributyltin (TBT), monophenyltin (MPT), diphenyltin (DPT), triphenyltin (TPT) and a surrogate standard (SS; ethyltri-*n*-propyltin).

| **Compound** | **Average recovery (%)** |
| --- | --- |
| MBT | 64.6 |
| DBT | 67.0 |
| TBT | 79.4 |
| MPT | 66.5 |
| DPT | 1.9 |
| TPT | 93.5 |
| SS | 73.4 |
