# Supplementary material for: Long-Term Spatio-Temporal Trends of Organotin Contaminations in the Marine Environment of Hong Kong
Source: PLoS One. 2016 May 13;11(5):e0155632. doi: 10.1371/journal.pone.0155632 (PMC4866715; doi:10.1371/journal.pone.0155632)
Supplement: S8 Table — (DOCX) [file pone.0155632.s008.docx]

**S8 Table. Method of evaluating risk quotient (RQ; also see Leung et al., 2006).**

| Measured tissue concentration (MTC) | MTCs of butyltins (BTs) and phenyltins (PTs) at each site were obtained from the present study, while the MTCs of BTs in 2004 were extracted from Leung et al. (2006). |
| --- | --- |
| Predicted no effect tissue concentration (PNETC) | Values of PNETC and bioconcentration factor (BCF) were extracted from peer-reviewed literatures, in which the PNETCs were derived from chronic and sub-lethal effects of BTs or PTs on molluscs (Table A.6 for BTs and Table A.7 for PTs, respectively). Values of predicted no effect concentration in water (PNEC) were converted to the respective tissue concentrations using appropriate BCFs, thus PNETC = PNEC x BCF. A geometric mean of PNETCs was obtained when there were more than one data from the same species. |
| Distributions of species’ sensitivity and tissue concentration | PNETCs were ranked and presented in cumulative distributions for BTs and PTs, respectively. MTCs for BTs and PTs were plotted as the exposure distribution in a cumulative function. All MTCs and PNETCs were standardized to ng BT or PT g^-1^ dw. |
