# Supplementary material for: Long-Term Spatio-Temporal Trends of Organotin Contaminations in the Marine Environment of Hong Kong
Source: PLoS One. 2016 May 13;11(5):e0155632. doi: 10.1371/journal.pone.0155632 (PMC4866715; doi:10.1371/journal.pone.0155632)
Supplement: S9 Table — (DOCX) [file pone.0155632.s009.docx]

**S9 Table. Chronic and sub-chronic toxicity values of body concentration of tributyltin (TBT; ng g^-1^ dry weight; arranged in ascending order) for molluscs, which were adopted from Appendix 4 of Leung et al. (2006) with modifications.** Values used in the construction of species sensitivity distribution (Fig 4a) were marked with asterisks (*) and bolded. N.A. means not available.

| **Taxa** | **Species** | **Chronic value**  **(ng TBT L^-1^)** | **Exposure duration and method** | **Biocon-**  **centration factor (BCF)** | **Estimated body concentration (ng g^-1^ dry weight as TBT)** | **Type of chronic value** | **End point of toxicity** |
| --- | --- | --- | --- | --- | --- | --- | --- |
| Gastropod | *Littorina littorea* | 4.9 ^a^ | Field study | 34600 ^b^ | 168.6 | LOEC | Female developed intersex ^c^ |
| Gastropod | *Nucella lapillus* | 2.4 ^a^ | Field study | 100000 ^d^ | 243.7 | LOEC | Females lost weight and developed imposex ^e^ |
| Gastropod | *Nucella lapillus* | 4.9 ^a^ | 8 days | 100000 ^d^ | 487.4 | LOEC | Females lost weight and developed imposex ^f^ |
|  |  |  |  |  | **271.56 *** | **Geometric mean of above three entries** | |
| Gastropod | *Reishia clavigera* ^v^ | N.A. | Field study | N.A. | 10−20 ng TBTCl g^-1^ wet tissue ^u^ | LOEC | Female developed imposex ^g^ |
| Gastropod | *Reishia clavigera* ^v^ | ca. 2.4 ^a^ | 85 days | 5000 – 10000 ^h^ | ca. 20 ng TBTCl g^-1^ wet tissue ^u^ | LOEC | Female developed imposex ^h^ |
| Gastropod | *Hinia incrassata* | 3.7 ^a^ | Field study | 122000 ^i^ | 446.0 | LOEC | Imposex development ^i^ |
| Bivalve | *Ruditapes decussata* | 219.3 ^a^ | 7 days exposure | 12000 ^j^ | **2631.9 *** | LOEC | Elevation in the NADPH cytochrome (P450) reductases and decrease in NADH cytochrome (b_5_) reductases ^k^ |
| Bivalve | *Crassostrea virginica* | 73.1 ^a^ | 9 weeks flow through experiment | 49000 ^l^ | **3582.4 *** | LOEC | Decrease resistance against bacterial challenge ^m^ |
| Bivalve | *Mytilus edulis* | 12.2 ^a^ | 32 days | 400000 ^n^ | 4915.3 | NOEC | Activity of hemocyte and membrane injury ^o^ |
| Bivalve | *Mytilus edulis* | 13.2 ^a^ | 15 days | 400000 ^n^ | 5263.9 | EC_10_ | Shell growth retardation ^p^ |
| Bivalve | *Mytilus edulis* | 20 | 60 days | 400000 ^n^ | 8000 | LOEC | Reduced shell length ^q^ |
|  |  |  |  |  | **5915.38 *** | **Geometric mean of above three entries** | |
| Bivalve | *Scrobicularia plana* | 121.8 ^a^ | 10 days exposure | 50000 ^r^ | **6092.4 *** | LOEC | Larvae reduced shell growth and survivorship ^s^ |
| Bivalve | *Crassostrea gigas* | 48.7 ^a^ | Field study | 310000 ^l^ | **15097 *** | LOEC | Growth inhibition ^t^ |

^a^ Converted to ng TBT L^-1^

^b^ Kure and Depledge (1994)

^c^ Oehlmann (2004)

^d^ Bryan et al. (1993)

^e^ Gibbs et al. (1987)

^f^ Davies et al. (1997)

^g^ Horiguchi et al. (1994)

^h^ Horiguchi et al. (1995)

^i^ Oehlmann et al. (1998)

^j^ Morcillo and Porte (2000)

^k^ Sole (2000)

^l^ Roberts et al. (1987)

^m^ Fisher et al. (1999)

^n^ Salazar and Salazar (1991)

^o^ St-Jean et al. (2002)

^p^ Stenalt et al. (1998)

^q^ Huang and Yong (1995)

^r^ Bryan and Gibbs (1991)

^s^ Ruiz et al. (2005)

^t^ Batley et al. (1989)

^u^ Values transformed into 33−67 and 67 ng TBTCl g^-1^ dry weight respectively, assuming the moisture content in the tissue was 70%.

^v^ Previously named as *Thais clavigera* (see Claremont et al., 2013)
