# Supplementary material for: Long-Term Spatio-Temporal Trends of Organotin Contaminations in the Marine Environment of Hong Kong
Source: PLoS One. 2016 May 13;11(5):e0155632. doi: 10.1371/journal.pone.0155632 (PMC4866715; doi:10.1371/journal.pone.0155632)
Supplement: S10 Table — (DOCX) [file pone.0155632.s010.docx]

**S10 Table. Chronic and sub-chronic toxicity values of body concentration of triphenyltin (TPT; ng g^-1^ dry weight; arranged in ascending order) for molluscs.** Values used in the construction of species sensitivity distribution (Fig 3a) were marked with asterisks (*) and bolded. N.A. means not available.

| **Taxa** | **Species** | **Chronic value**  **(ng TPT L^-1^)** | **Exposure duration and method** | **Biocon-**  **centration factor (BCF)** | **Estimated body concentration (ng g^-1^ dry weight as TPT)** | **Type of chronic value** | **End point of toxicity** |
| --- | --- | --- | --- | --- | --- | --- | --- |
| Gastropod | *Reishia clavigera* ^r^ | N.A. | Field study | N.A. | 52.7 ^j^ | LOEC | Females developed imposex ^a^ |
| Gastropod | *Reishia clavigera* ^r^ | N.A. | Field study | N.A. | 8 | LOEC | Females developed imposex ^b^ |
| Gastropod | *Reishia clavigera* ^r^ | N.A. | Field study | N.A. | 34.9 | LOEC | Females developed imposex ^c^ |
|  |  |  |  |  | **24.50 *** | **Geometric mean of above three entries** | |
| Gastropod | *Reishia clavigera* ^r^ | N.A. | Field study | N.A. | **114.6 ^j^ *** | LOEC | Elevated RXR gene expression ^d^ |
| Gastropod | *Haliotis gigantea* | 100 | 63 days flow- through exposure | 31623 ^k^ | **3162.3 *** | LOEC | Ovarian spermatogenesis; increased in numbers of contracted primary oocytes ^e^ |
| Gastropod | *Haliotis madaka* | N.A. | Field study | N.A. | 9.0 ^l^ | LOEC | Masculinization of females ^f^ |
| Gastropod | *Babylonia japonica* | N.A. | Field study | N.A. | 1206.6 ^m^ | LOEC | Females developed imposex ^g^ |
| Gastropod | *Bolinus brandaris* | N.A. | Field study | N.A. | 13.1 ^n^ | LOEC | Females developed imposex ^a^ |
| Gastropod | *Marisa cornuarietis* | 88.5 ^o^ | 7 days | 31623 ^k^ | 2798.6 | LOEC | Increase in esterified testosterone ^h^ |
| Gastropod | *Marisa cornuarietis* | 1474.3 ^o^ | 7 days | 31623 ^k^ | 46621.8 | LOEC | Decrease in esterified estradiol ^h^ |
| Gastropod | *Marisa cornuarietis* | 36.3 ^o^ | 4 months | 31623 ^k^ | 1147.9 | EC10 | Females developed imposex ^i^ |
| Gastropod | *Marisa cornuarietis* | 16.5 ^o^ | 4 months | 31623 ^k^ | 521.8 | EC10 | Reduced fecundity ^i^ |
| Gastropod | *Marisa cornuarietis* | 737.1 ^o^ | 4 months | 31623 ^k^ | 23309.3 | LOEC | Spawning inhibition ^i^ |
| Gastropod | *Marisa cornuarietis* | 221.1 ^o^ | 2 months | 31623 ^k^ | 6991.8 | LOEC | Reduced spermatogenesis ^i^ |
|  |  |  |  |  | **4832.50 *** | **Geometric mean of above six entries** | |
| Gastropod | *Nucella lapillus* | 294.9 ^o^ | 4 months | 100000 ^p^ | **29490 *** | LOEC | Reduced pallial sex organs in males ^i^ |
| Gastropod | *Hinia reticulata* | 147.4 ^o^ | 1 month | 11000 ^q^ | **1621.4 *** | LOEC | Retarded follicle maturation in females ^i^ |

^a^ Horiguchi et al. (1997)

^b^ Shim et al. (2000)

^c^ Hung et al. (2001)

^d^ Horiguchi et al. (2007)

^e^ Horiguchi et al. (2002)

^f^ Horiguchi et al. (2000)

^g^ Horiguchi (2006)

^h^ Lyssimachou et al. (2008)

^i^ Schulte-Oehlmann et al. (2000)

^j^ Assuming the moisture content of the whole soft tissues of *Reishia clavigera* (*Thais clavigera*) is 70%, the value has been translated for dry weight.

^k^ Estimated BCF from geometric mean of 10000 and 100000

^l^ Assuming the moisture content of the whole soft tissues of *Haliotis madaka* is 70%, the value has been translated for dry weight.

^m^ Measured in females

^n^ Assuming the moisture content of the whole soft tissues of *Bolinus brandaris* is 70%, the value has been translated for dry weight.

^o^ Converted as ng TPT L^-1^

^p^ Bryan et al. (1993)

^q^ Schulte-Oehlmann et al. (1998); BCF for TBT

^r^ Previously named as *Thais clavigera* (see Claremont et al., 2013)
