# Supplementary material for: Long-Term Spatio-Temporal Trends of Organotin Contaminations in the Marine Environment of Hong Kong
Source: PLoS One. 2016 May 13;11(5):e0155632. doi: 10.1371/journal.pone.0155632 (PMC4866715; doi:10.1371/journal.pone.0155632)
Supplement: S11 Table — (DOCX) [file pone.0155632.s011.docx]

**S11 Table. Tissue concentrations (in μg kg^-1^ dry weight) of six organotins (OTs): monobutyltin (MBT), dibutyltin (DBT), tributyltin (TBT), monophenyltin (MPT), diphenyltin (DPT) and triphenyltin (TPT) in *Reishia clavigera* collected in 2004–06 (site 1–27 from Leung et al. (2006) and site 28–29 from Qiu et al. (2011, summer data only)), 2010 and 2015 from Hong Kong.** Total butyltins (total BTs; sum of MBT, DBT and TBT), total phenyltins (total PTs; sum of MPT, DPT and TPT) and total OTs (sum of all six OTs) were listed. All values have been transformed into the unit of μg kg^-1^ dry weight. N.A. means data not available, N.E. means not evaluated and B.D.L. means data below detection limit.

| **No.** | **Site** | **2004–06** | | | |  | **2010** |  |  |  |  |  |  |  |  |  |  | **2015** | | | | | | | | | |  |
| --- | --- | --- | --- | --- | --- | --- | --- | --- | --- | --- | --- | --- | --- | --- | --- | --- | --- | --- | --- | --- | --- | --- | --- | --- | --- | --- | --- | --- |
|  |  | **MBT** | **DBT** | **TBT** | **Total BTs** |  | **MBT** | **DBT** | **TBT** | **Total BTs** | **MPT** | **DPT** | **TPT** | **Total PTs** |  | **Total OTs** |  | **MBT** | | **DBT** | **TBT** | **Total BTs** | **MPT** | **DPT** | **TPT** | **Total PTs** | **Total OTs** | |
| 1 | Kat O | 44.8 | 63.6 | 22.7 | 131.1 |  | 6.4 | 21.5 | 9.5 | 37.4 | 88.4 | 17.0 | 1077.6 | 1183.0 |  | 1220.4 |  | N.E. | | | | | | | | | | |
| 2 | Pak Sha Chau | 31.3 | 39.8 | 20.8 | 91.9 |  | 5.9 | 21.1 | 16.0 | 43.1 | 41.7 | 16.3 | 1195.2 | 1253.2 |  | 1296.3 |  | N.E. | | | | | | | | | | |
| 3 | Chek Chau | B.D.L. | B.D.L. | B.D.L. | B.D.L. |  | 5.1 | 18.8 | 8.0 | 31.9 | 34.0 | 268.0 | 279.5 | 581.6 |  | 613.5 |  | N.E. | | | | | | | | | | |
| 4 | Wu Kwai Sha | 159.5 | B.D.L. | 9.3 | > 168.8 |  | 3.8 | 25.7 | 12.3 | 41.7 | 24.0 | 25.9 | 2164.7 | 2214.6 |  | 2256.4 |  | N.E. | | | | | | | | | | |
| 5 | Heng On | B.D.L. | 33.2 | B.D.L. | > 33.2 |  | N.A. | | | | | | | | | |  |  | N.A. | | | | | | | | |  |
| 6 | Wong Mau Chau | 106.5 | 23.9 | 4.4 | 134.8 |  | 6.0 | 13.2 | 11.3 | 30.6 | 55.8 | 22.6 | 2603.1 | 2681.4 |  | 2712.0 |  | N.E. | | | | | | | | | | |
| 7 | Kong Tau Pai | 48.8 | 12.4 | 4.4 | 65.5 |  | 3.9 | 14.9 | 5.8 | 24.6 | 31.3 | 18.8 | 541.2 | 591.2 |  | 615.8 |  | N.E. | | | | | | | | | | |
| 8 | Sai Kung Pier | 65.8 | 108.0 | 36.2 | 209.9 |  | 4.8 | 13.5 | 47.1 | 65.4 | 76.5 | 29.0 | 11108.0 | 11213.5 |  | 11278.9 |  | 58.2 | | 23.5 | 6.3 | 88.0 | 64.9 | 25.3 | 15059.6 | 15149.8 | 15304.9 | |
| 9 | UST | 24.0 | 36.5 | 12.2 | 72.7 |  | 7.5 | 18.1 | 28.1 | 53.7 | 92.5 | 25.3 | 4216.8 | 4334.7 |  | 4388.4 |  | N.E. | | | | | | | | | | |
| 10 | Clear Water Bay | B.D.L. | B.D.L. | B.D.L. | N.A. |  | 1.4 | 10.4 | 10.3 | 22.1 | 14.0 | 27.5 | 457.8 | 499.3 |  | 521.4 |  | 12.6 | | 22.5 | B.D.L. | 35.3 | 40.8 | B.D.L. | 701.1 | 742.3 | 777.6 | |
| 11 | Shek Mei Tao | 33.4 | 39.1 | B.D.L. | > 72.5 |  | 6.3 | 16.7 | 16.6 | 39.5 | 34.4 | 26.5 | 1951.9 | 2012.7 |  | 2052.3 |  | N.E. | | | | | | | | | | |
| 12 | Tung Lung Island | 61.1 | 56.9 | 14.2 | 132.2 |  | 2.7 | 8.7 | 19.8 | 31.2 | 69.2 | 60.4 | 881.3 | 1011.0 |  | 1042.2 |  | N.E. | | | | | | | | | | |
| 13 | Waglan Island | 17.8 | 11.0 | 5.9 | 34.6 |  | 2.6 | 9.1 | 17.5 | 29.2 | 29.8 | 29.0 | 498.0 | 556.9 |  | 586.2 |  | N.E. | | | | | | | | | | |
| 14 | Po Toi | 56.0 | 34.5 | 9.8 | 100.4 |  | 10.0 | 22.0 | 52.9 | 84.8 | 48.6 | 31.8 | 1482.4 | 1562.8 |  | 1647.7 |  | 18.0 | | 21.2 | 17.2 | 56.4 | 27.8 | B.D.L. | 1079.1 | 1107.4 | 1163.9 | |
| 15 | Shek O | B.D.L. | B.D.L. | 10.5 | > 10.5 |  | 4.1 | 14.6 | 22.1 | 40.8 | 18.8 | 31.0 | 227.9 | 277.7 |  | 318.5 |  | B.D.L. | | B.D.L. | 4.4 | 5.5 | 25.7 | B.D.L. | 612.4 | 638.6 | 643.9 | |
| 16 | Turtle Cove | 40.5 | 12.6 | 14.7 | 67.7 |  | 4.5 | 14.0 | 23.2 | 41.7 | 52.7 | 44.8 | 3081.7 | 3179.2 |  | 3220.9 |  | 12.9 | | 27.1 | B.D.L. | 40.1 | 23.3 | 1.8 | 1264.6 | 1289.8 | 1329.9 | |
| 17 | Chung Hum Kok | 31.6 | 25.9 | 7.8 | 65.3 |  | 14.7 | 67.1 | 38.1 | 120.0 | 87.3 | 45.0 | 6133.4 | 6265.7 |  | 6385.7 |  | N.E. | | | | | | | | | | |
| 18 | Repulse Bay | 499.0 | 64.8 | 24.2 | 588.0 |  | 10.3 | 21.7 | 35.7 | 67.6 | 35.5 | 14.6 | 456.6 | 506.6 |  | 574.2 |  | N.E. | | | | | | | | | | |
| 19 | Deep Water Bay | 41.7 | 65.9 | 32.0 | 139.6 |  | 10.1 | 25.3 | 44.7 | 80.1 | 40.3 | 30.9 | 1244.3 | 1315.5 |  | 1395.6 |  | 16.5 | | 21.2 | 23.0 | 60.7 | 49.0 | B.D.L. | 2589.9 | 2639.4 | 2700.0 | |
| 20 | Aberdeen | 1038.9 | 387.3 | 40.8 | 1466.9 |  | 12.1 | 33.1 | 51.5 | 96.7 | 78.6 | 43.5 | 3751.0 | 3873.0 |  | 3969.7 |  | 137.6 | | 48.7 | 38.2 | 224.5 | 63.4 | 17.4 | 8607.1 | 8687.9 | 8912.3 | |
| 21 | Sok Kwu Wan | 57.5 | 96.2 | 30.3 | 184.0 |  | 9.2 | 25.4 | 90.8 | 125.4 | 56.4 | 32.4 | 7315.2 | 7404.0 |  | 7529.4 |  | 85.6 | | 24.8 | 20.5 | 130.9 | 80.6 | 5.4 | 3644.6 | 3730.6 | 3861.5 | |
| 22 | Ha Mei Wan | 160.7 | 77.3 | 44.7 | 282.8 |  | 0.8 | 5.3 | 16.2 | 22.4 | 14.7 | 19.8 | 579.8 | 614.3 |  | 636.7 |  | N.E. | | | | | | | | | | |
| 23 | Mui Wo | 40.3 | 283.0 | 26.6 | 350.0 |  | 8.0 | 38.5 | 43.0 | 89.5 | 33.6 | 31.5 | 1731.3 | 1796.4 |  | 1885.9 |  | N.E. | | | | | | | | | | |
| 24 | Cheung Sha | 21.1 | 70.1 | 14.2 | 105.3 |  | 3.1 | 10.7 | 38.2 | 52.0 | 35.0 | 22.2 | 1075.2 | 1132.5 |  | 1184.5 |  | N.E. | | | | | | | | | | |
| 25 | Tai O | 31.1 | 111.5 | 38.6 | 181.2 |  | 3.2 | 24.6 | 39.0 | 66.8 | 17.0 | 26.0 | 635.2 | 678.2 |  | 744.9 |  | N.E. | | | | | | | | | | |
| 26 | Butterfly Beach | 63.2 | 258.5 | 44.7 | 366.4 |  | 8.1 | 36.0 | 240.1 | 284.2 | 68.6 | 30.1 | 1951.8 | 2050.5 |  | 2334.8 |  | 37.0 | | 48.4 | 117.3 | 202.7 | 30.6 | 8.6 | 7467.6 | 7506.8 | 7709.4 | |
| 27 | Kadoorie Beach | 60.9 | 114.8 | 37.6 | 213.4 |  | 7.5 | 52.2 | 422.0 | 481.7 | 35.0 | 15.5 | 2373.2 | 2423.7 |  | 2905.4 |  | 66.0 | | 49.4 | 112.9 | 228.4 | 26.8 | 6.3 | 5198.5 | 5231.6 | 5460.0 | |
| 28 | Pak Sha Wan | 18.0 | 125.1 | 59.2 | 202.3 |  | 1.7 | 18.4 | 49.3 | 69.4 | 18.0 | 39.3 | 1610.5 | 1667.8 |  | 1737.2 |  | N.E. | | | | | | | | | | |
| 29 | Waterfall Bay | 7.8 | 102.9 | 53.3 | 164.1 |  | 17.8 | 47.3 | 147.6 | 212.7 | 113.5 | 54.3 | 4359.5 | 4527.4 |  | 4740.1 |  | N.E. | | | | | | | | | | |
