# Supplementary material for: Long-Term Spatio-Temporal Trends of Organotin Contaminations in the Marine Environment of Hong Kong
Source: PLoS One. 2016 May 13;11(5):e0155632. doi: 10.1371/journal.pone.0155632 (PMC4866715; doi:10.1371/journal.pone.0155632)
Supplement: S12 Table — (DOCX) [file pone.0155632.s012.docx]

**S12 Table. Tissue concentrations (in μg Sn kg^-1^ wet weight) of six organotins (OTs): monobutyltin (MBT), dibutyltin (DBT), tributyltin (TBT), monophenyltin (MPT), diphenyltin (DPT) and triphenyltin (TPT) in *Reishia clavigera* collected in 2004–06 (site 1–27 from Leung et al. (2006) and site 28–29 from Qiu et al. (2011, summer data only)), 2010 and 2015 from Hong Kong.** Total butyltins (total BTs; sum of MBT, DBT and TBT), total phenyltins (total PTs; sum of MPT, DPT and TPT) and total OTs (sum of all six OTs) were listed. N.A. means data not available, N.E. means not evaluated and B.D.L. means data below detection limit.

| **No.** | **Site** | **2004–06** | | | |  | **2010** |  |  |  |  |  |  |  |  |  |  | **2015** | | | | | | | | | |  |
| --- | --- | --- | --- | --- | --- | --- | --- | --- | --- | --- | --- | --- | --- | --- | --- | --- | --- | --- | --- | --- | --- | --- | --- | --- | --- | --- | --- | --- |
|  |  | **MBT** | **DBT** | **TBT** | **Total BTs** |  | **MBT** | **DBT** | **TBT** | **Total BTs** | **MPT** | **DPT** | **TPT** | **Total PTs** |  | **Total OTs** |  | **MBT** | | **DBT** | **TBT** | **Total BTs** | **MPT** | **DPT** | **TPT** | **Total PTs** | **Total OTs** | |
| 1 | Kat O | 6.0 | 6.5 | 1.9 | 14.4 |  | 0.9 | 2.2 | 0.8 | 3.8 | 10.7 | 1.5 | 72.9 | 85.0 |  | 88.8 |  | N.E. | | | | | | | | | | |
| 2 | Pak Sha Chau | 4.2 | 4.1 | 1.7 | 10.0 |  | 0.8 | 2.2 | 1.3 | 4.3 | 5.0 | 1.4 | 80.8 | 87.3 |  | 91.5 |  | N.E. | | | | | | | | | | |
| 3 | Chek Chau | B.D.L. | B.D.L. | B.D.L. | B.D.L. |  | 0.7 | 1.9 | 0.7 | 3.3 | 4.1 | 23.2 | 18.9 | 46.2 |  | 49.5 |  | N.E. | | | | | | | | | | |
| 4 | Wu Kwai Sha | 21.5 | B.D.L. | 0.8 | > 22.3 |  | 0.5 | 2.6 | 1.0 | 4.1 | 2.9 | 2.2 | 146.4 | 151.6 |  | 155.7 |  | N.E. | | | | | | | | | | |
| 5 | Heng On | B.D.L. | 3.4 | B.D.L. | > 3.4 |  | N.A. | | | | | | | | | |  |  | N.A. | | | | | | | | |  |
| 6 | Wong Mau Chau | 14.4 | 2.4 | 0.4 | 17.2 |  | 0.8 | 1.3 | 0.9 | 3.1 | 6.7 | 2.0 | 176.1 | 184.8 |  | 187.8 |  | N.E. | | | | | | | | | | |
| 7 | Kong Tau Pai | 6.6 | 1.3 | 0.4 | 8.2 |  | 0.5 | 1.5 | 0.5 | 2.5 | 3.8 | 1.6 | 36.6 | 42.0 |  | 44.5 |  | N.E. | | | | | | | | | | |
| 8 | Sai Kung Pier | 8.9 | 11.0 | 3.0 | 22.8 |  | 0.6 | 1.4 | 3.9 | 5.9 | 9.2 | 2.5 | 751.4 | 763.1 |  | 769.0 |  | 7.9 | | 2.4 | 0.5 | 10.8 | 7.8 | 2.2 | 1018.6 | 1028.7 | 1039.4 | |
| 9 | UST | 3.2 | 3.7 | 1.0 | 8.0 |  | 1.0 | 1.8 | 2.3 | 5.2 | 11.1 | 2.2 | 285.2 | 298.6 |  | 303.7 |  | N.E. | | | | | | | | | | |
| 10 | Clear Water Bay | B.D.L. | B.D.L. | B.D.L. | B.D.L. |  | 0.2 | 1.1 | 0.8 | 2.1 | 1.7 | 2.4 | 31.0 | 35.0 |  | 37.1 |  | 1.7 | | 2.3 | B.D.L. | 4.2 | 4.9 | B.D.L. | 47.4 | 52.8 | 57.0 | |
| 11 | Shek Mei Tao | 4.5 | 4.0 | B.D.L. | > 8.5 |  | 0.8 | 1.7 | 1.4 | 3.9 | 4.1 | 2.3 | 132.0 | 138.5 |  | 142.4 |  | N.E. | | | | | | | | | | |
| 12 | Tung Lung Island | 8.2 | 5.8 | 1.2 | 15.2 |  | 0.4 | 0.9 | 1.6 | 2.9 | 8.3 | 5.2 | 59.6 | 73.2 |  | 76.1 |  | N.E. | | | | | | | | | | |
| 13 | Waglan Island | 2.4 | 1.1 | 0.5 | 4.0 |  | 0.4 | 0.9 | 1.4 | 2.7 | 3.6 | 2.5 | 33.7 | 39.8 |  | 42.5 |  | N.E. | | | | | | | | | | |
| 14 | Po Toi | 7.6 | 3.5 | 0.8 | 11.9 |  | 1.3 | 2.2 | 4.3 | 7.9 | 5.9 | 2.8 | 100.3 | 108.9 |  | 116.8 |  | 2.4 | | 2.2 | 1.4 | 6.0 | 3.4 | B.D.L. | 73.0 | 76.8 | 82.8 | |
| 15 | Shek O | B.D.L. | B.D.L. | 0.9 | > 0.9 |  | 0.6 | 1.5 | 1.8 | 3.9 | 2.3 | 2.7 | 15.4 | 20.4 |  | 24.2 |  | B.D.L. | | B.D.L. | 0.4 | 1.5 | 3.1 | B.D.L. | 41.4 | 45.0 | 46.5 | |
| 16 | Turtle Cove | 5.5 | 1.3 | 1.2 | 8.0 |  | 0.6 | 1.4 | 1.9 | 3.9 | 6.4 | 3.9 | 208.4 | 218.7 |  | 222.6 |  | 1.7 | | 2.8 | B.D.L. | 4.7 | 2.8 | 0.2 | 85.5 | 88.5 | 93.2 | |
| 17 | Chung Hum Kok | 4.3 | 2.6 | 0.6 | 7.5 |  | 2.0 | 6.8 | 3.1 | 11.9 | 10.5 | 3.9 | 414.9 | 429.3 |  | 441.2 |  | N.E. | | | | | | | | | | |
| 18 | Repulse Bay | 67.3 | 6.6 | 2.0 | 75.9 |  | 1.4 | 2.2 | 2.9 | 6.5 | 4.3 | 1.3 | 30.9 | 36.4 |  | 43.0 |  | N.E. | | | | | | | | | | |
| 19 | Deep Water Bay | 5.6 | 6.7 | 2.6 | 15.0 |  | 1.4 | 2.6 | 3.7 | 7.6 | 4.9 | 2.7 | 84.2 | 91.7 |  | 99.3 |  | 2.2 | | 2.2 | 1.9 | 6.3 | 5.9 | B.D.L. | 175.2 | 181.1 | 187.4 | |
| 20 | Aberdeen | 140.1 | 39.5 | 3.3 | 182.9 |  | 1.6 | 3.4 | 4.2 | 9.2 | 9.5 | 3.8 | 253.7 | 267.0 |  | 276.2 |  | 18.6 | | 5.0 | 3.1 | 26.7 | 7.6 | 1.5 | 582.2 | 591.3 | 618.0 | |
| 21 | Sok Kwu Wan | 7.8 | 9.8 | 2.5 | 20.0 |  | 1.2 | 2.6 | 7.4 | 11.3 | 6.8 | 2.8 | 494.8 | 504.4 |  | 515.7 |  | 11.5 | | 2.5 | 1.7 | 15.8 | 9.7 | 0.5 | 246.5 | 256.7 | 272.5 | |
| 22 | Ha Mei Wan | 21.7 | 7.9 | 3.7 | 33.2 |  | 0.1 | 0.5 | 1.3 | 2.0 | 1.8 | 1.7 | 39.2 | 42.7 |  | 44.7 |  | N.E. | | | | | | | | | | |
| 23 | Mui Wo | 5.4 | 28.8 | 2.2 | 36.4 |  | 1.1 | 3.9 | 3.5 | 8.5 | 4.0 | 2.7 | 117.1 | 123.9 |  | 132.4 |  | N.E. | | | | | | | | | | |
| 24 | Cheung Sha | 2.8 | 7.1 | 1.2 | 11.2 |  | 0.4 | 1.1 | 3.1 | 4.6 | 4.2 | 1.9 | 72.7 | 78.9 |  | 83.5 |  | N.E. | | | | | | | | | | |
| 25 | Tai O | 4.2 | 11.4 | 3.2 | 18.7 |  | 0.4 | 2.5 | 3.2 | 6.1 | 2.0 | 2.3 | 43.0 | 47.3 |  | 53.4 |  | N.E. | | | | | | | | | | |
| 26 | Butterfly Beach | 8.5 | 26.3 | 3.7 | 38.5 |  | 1.1 | 3.7 | 19.7 | 24.4 | 8.3 | 2.6 | 132.0 | 142.9 |  | 167.3 |  | 5.0 | | 4.9 | 9.6 | 19.5 | 3.7 | 0.7 | 505.1 | 509.5 | 529.1 | |
| 27 | Kadoorie Beach | 8.2 | 11.7 | 3.1 | 23.0 |  | 1.0 | 5.3 | 34.5 | 40.9 | 4.2 | 1.3 | 160.5 | 166.1 |  | 207.0 |  | 8.9 | | 5.0 | 9.2 | 23.2 | 3.2 | 0.5 | 351.6 | 355.4 | 378.6 | |
| 28 | Pak Sha Wan | 2.4 | 12.7 | 4.8 | 20.0 |  | 0.2 | 1.9 | 4.0 | 6.1 | 2.2 | 3.4 | 108.9 | 114.5 |  | 120.7 |  | N.E. | | | | | | | | | | |
| 29 | Waterfall Bay | 1.1 | 10.5 | 4.4 | 15.9 |  | 2.4 | 4.8 | 12.1 | 19.3 | 13.7 | 4.7 | 294.9 | 313.3 |  | 332.6 |  | N.E. | | | | | | | | | | |
