# Supplementary material for: Long-Term Spatio-Temporal Trends of Organotin Contaminations in the Marine Environment of Hong Kong
Source: PLoS One. 2016 May 13;11(5):e0155632. doi: 10.1371/journal.pone.0155632 (PMC4866715; doi:10.1371/journal.pone.0155632)
Supplement: S13 Table — (DOCX) [file pone.0155632.s013.docx]

**S13 Table. Degradation indices (Butyltin Degradation Index, BDI and Phenyltin Degradation Index, PDI) of butyltin (Leung et al., 2006; Qiu et al., 2010 and this study) and phenyltin compounds (this study only) in the tissues of *Reishia clavigera*.** N.A. means not data available and N.E. means not evaluated.

| No. | Site | 2004–06 |  | 2010 |  |  | 2015 |  |
| --- | --- | --- | --- | --- | --- | --- | --- | --- |
|  |  | BDI |  | BDI ^a^ | PDI ^b^ |  | BDI ^a^ | PDI ^b^ |
| 1 | Kat O | 4.77 ^c^ |  | 2.92 | 0.10 |  | N.E. | N.E. |
| 2 | Pak Sha Chau | 3.43 ^c^ |  | 1.69 | 0.05 |  | N.E. | N.E. |
| 3 | Chek Chau | N.E. |  | 2.99 | 1.11 |  | N.E. | N.E. |
| 4 | Wu Kai Sha | 17.19 ^c,e^ |  | 2.39 | 0.02 |  | N.E. | N.E. |
| 5 | Heng On | 388.56 ^c,e^ |  | N.A. | N.A. |  | N.E. | N.E. |
| 6 | Wong Mau Chau | 29.65 ^c^ |  | 1.70 | 0.03 |  | N.E. | N.E. |
| 7 | Kong Tau Pai | 13.90 ^c^ |  | 3.25 | 0.09 |  | N.E. | N.E. |
| 8 | Sai Kung Pier | 4.81 ^c^ |  | 0.39 | 0.01 |  | 12.95 | 0.01 |
| 9 | UST | 4.96 ^c^ |  | 0.91 | 0.03 |  | N.E. | N.E. |
| 10 | Clear Water Bay | N.E. |  | 1.15 | 0.09 |  | 175.53 | 0.06 |
| 11 | Shek Mei Tao | 846.97 ^c,e^ |  | 1.38 | 0.03 |  | N.E. | N.E. |
| 12 | Tung Lung Island | 8.33 ^c^ |  | 0.58 | 0.15 |  | N.E. | N.E. |
| 13 | Waglan Island | 4.91 ^c^ |  | 0.67 | 0.12 |  | N.E. | N.E. |
| 14 | Po Toi | 9.27 ^c^ |  | 0.60 | 0.05 |  | 2.29 | 0.03 |
| 15 | Shek O | 0.01 ^c,e^ |  | 0.85 | 0.22 |  | 0.26 | 0.04 |
| 16 | Turtle Cove | 3.62 ^c^ |  | 0.80 | 0.03 |  | 199.57 | 0.02 |
| 17 | Chung Hum Kok | 7.35 ^c^ |  | 2.15 | 0.02 |  | N.E. | N.E. |
| 18 | Repulse Bay | 23.31 ^c^ |  | 0.90 | 0.11 |  | N.E. | N.E. |
| 19 | Deep Water Bay | 3.36 ^c^ |  | 0.79 | 0.06 |  | 1.64 | 0.02 |
| 20 | Aberdeen | 34.96 ^c^ |  | 0.88 | 0.03 |  | 4.87 | 0.01 |
| 21 | Sok Kwu Wan | 5.07 ^c^ |  | 0.38 | 0.01 |  | 5.37 | 0.02 |
| 22 | Ha Mei Wan | 5.32 ^c^ |  | 0.38 | 0.06 |  | N.E. | N.E. |
| 23 | Mui Wo | 12.14 ^c^ |  | 1.08 | 0.04 |  | N.E. | N.E. |
| 24 | Cheung Sha | 6.43 ^c^ |  | 0.36 | 0.05 |  | N.E. | N.E. |
| 25 | Tai O | 3.69 ^c^ |  | 0.71 | 0.07 |  | N.E. | N.E. |
| 26 | Butterfly Beach | 7.19 ^c^ |  | 0.18 | 0.10 |  | 0.73 | 0.01 |
| 27 | Kadoorie Beach | 4.67 ^c^ |  | 0.14 | 0.05 |  | 1.02 | 0.01 |
| 28 | Pak Sha Wan | 2.41 ^d^ |  | 0.41 | 1.11 |  | N.E. | N.E. |
| 29 | Waterfall Bay | 2.08 ^d^ |  | 0.44 | 0.02 |  | N.E. | N.E. |

^a^ BDI = [MBT]+[DBT]/[TBT]

^b^ PDI = [MPT]+[DPT]/[TPT]

^c^ Data obtained from Leung et al. (2006)

^d^ Data obtained from Qiu et al. (2010); organotin concentration combined by considering the sex ratio.

^e^ Half of the detection limits were used in the calculations.
