# Supplementary material for: Long-Term Spatio-Temporal Trends of Organotin Contaminations in the Marine Environment of Hong Kong
Source: PLoS One. 2016 May 13;11(5):e0155632. doi: 10.1371/journal.pone.0155632 (PMC4866715; doi:10.1371/journal.pone.0155632)
Supplement: S15 Table — (DOCX) [file pone.0155632.s015.docx]

**S15 Table. Results of Spearman’s rank correlation analyses among tissue concentrations of total organotins (total OTs), triphenyltin (TPT), diphenyltin (DPT), tributyltin (TBT), Relative Penis Size Index (RPSI), median Vas Deferens Sequence Index (VDSI), mean VDSI and condition index (CI) in *Reishia clavigera* collected in 2015.** Values of *r_s_* (upper row) and *p* (lower row) are shown in the table. Significant correlations after sequential Bonferroni correction are marked with asterisks (*).

|  | Total OTs | TPT | DPT | TBT | RPSI | Median VDSI | Mean VDSI |
| --- | --- | --- | --- | --- | --- | --- | --- |
| TPT | *r_s_* > 0.999  *p* < 0.001 * |  |  |  |  |  |  |
| DPT | *r_s_* = 0.938  *p* < 0.001 * | *r_s_* = 0.938  *p* < 0.001 * |  |  |  |  |  |
| TBT | *r_s_* = 0.614  *p* = 0.059 | *r_s_* = 0.614  *p* = 0.059 | *r_s_* = 0.467  *p* = 0.173 |  |  |  |  |
| RPSI | *r_s_* = 0.855  *p* = 0.002 * | *r_s_* = 0.855  *p* = 0.002 * | *r_s_* = 0.807  *p* = 0.005 | *r_s_* = 0.644  *p* = 0.044 |  |  |  |
| Median VDSI | *r_s_* = 0.629  *p* = 0.051 | *r_s_* = 0.629  *p* = 0.051 | *r_s_* = 0.485  *p* = 0.156 | *r_s_* = 0.754  *p* = 0.012 | *r_s_* = 0.787  *p* = 0.007 |  |  |
| Mean VDSI | *r_s_* = 0.600  *p* = 0.067 | *r_s_* = 0.600  *p* = 0.067 | *r_s_* = 0.506  *p* = 0.135 | *r_s_* = 0.523  *p* = 0.121 | *r_s_* = 0.636  *p* = 0.048 | *r_s_* = 0.809  *p* = 0.005 |  |
| CI | *r_s_* = 0.612  *p* = 0.060 | *r_s_* = 0.612  *p* = 0.060 | *r_s_* = 0.557  *p* = 0.095 | *r_s_* = 0.754  *p* = 0.012 | *r_s_* = 0.685  *p* = 0.029 | *r_s_* = 0.787  *p* = 0.007 | *r_s_* = 0.673  *p* = 0.033 |
