# Supplementary material for: Long-Term Spatio-Temporal Trends of Organotin Contaminations in the Marine Environment of Hong Kong
Source: PLoS One. 2016 May 13;11(5):e0155632. doi: 10.1371/journal.pone.0155632 (PMC4866715; doi:10.1371/journal.pone.0155632)
Supplement: S16 Table — (DOCX) [file pone.0155632.s016.docx]

**S16 Table. Spearman’s rank correlation analyses between the distance to major shipping activities and imposex status (including Vas Deferens Sequence Index (VDSI) and Relative Penis Size Index (RPSI)), condition index and tissue concentrations of organotins.** Significant relationships, after sequential Bonferroni correction, are marked with asterisks.

|  | **2010** | **2015** |
| --- | --- | --- |
|  | **Distance to major shipping activities** (*n* = 28) | **Distance to major shipping activities** (*n* = 10) |
| **Mean VDSI** | *r_s_* = -0.744, *p* < 0.001 * | *r_s_* = -0.661, *p* = 0.038 |
| **Median VDSI** | *r_s_* = -0.701, *p* < 0.001 * | *r_s_* = -0.674, *p* = 0.033 |
| **RPSI** | *r_s_* = -0.783, *p* < 0.001 * | *r_s_* = -0.830, *p* = 0.003 * |
| **% sterile female** | *r_s_* = -0.703, *p* < 0.001 * | *r_s_* = -0.699, *p* = 0.024 |
| **Condition index** | *r_s_* = -0.552, *p* = 0.002 * | *r_s_* = -0.491, *p* = 0.150 |
| **Tissue concentration of total OTs** | *r_s_* = -0.423, *p* = 0.025 | *r_s_* = -0.806, *p* = 0.005 * |
| **Tissue concentration of total BTs** | *r_s_* = -0.639, *p* < 0.001 * | *r_s_* = -0.697, *p* = 0.025 |
| **Tissue concentration of TBT** | *r_s_* = -0.632, *p* < 0.001 * | *r_s_* = -0.474, *p* = 0.166 |
| **Tissue concentration of total PTs** | *r_s_* = -0.408, *p* = 0.031 | *r_s_* = -0.806, *p* = 0.005 * |
| **Tissue concentration of TPT** | *r_s_* = -0.402, *p* = 0.034 | *r_s_* = -0.806, *p* = 0.005 * |
| **Tissue concentration of DPT** | *r_s_* = -0.058, *p* = 0.768 | *r_s_* = -0.794, *p* = 0.006 * |

OTs: organotin; BTs: butyltins; TBT: tributyltin; PTs: phenyltins; TPT: triphenyltin; DPT: diphenyltin.
