# Supplementary material for: Long-Term Spatio-Temporal Trends of Organotin Contaminations in the Marine Environment of Hong Kong
Source: PLoS One. 2016 May 13;11(5):e0155632. doi: 10.1371/journal.pone.0155632 (PMC4866715; doi:10.1371/journal.pone.0155632)
Supplement: S17 Table — (DOCX) [file pone.0155632.s017.docx]

**S17 Table. List of references of Supporting Information (S1–S16 Tables).**

| Batley, G.E., Fuhua, C., Brockbank, C.I., Flegg, K.J., 1989. Accumulation of tributyltin by the Sydney rock oyster, *Saccostrea commercialis*. Aus. J. Mar. Freshwater Res. 40, 49–54.  Bryan, G.W., Bright, D.A., Hummerstone, L.G., Burt, G.R., 1993. Uptake, tissue distribution and metabolism of super(14)C-labelled tributyltin (TBT) in the dog-whelk, *Nucella lapillus*. J. Mar. Biol. Assoc. UK 73, 889–912.  Bryan, G.W., Gibbs, P.E., 1991. Impact of low concentrations of tributyltin (TBT) on marine organisms: a review. In: Newman, M.C., McIntosh, A.W. (Eds.), Metal Ecotoxicology: Concepts & Applications. Lewis Publishers, Michigan, pp. 323–361.  Cheung, M.S., Leung, H.Y.M., Leung, K.M.Y., 2010. The Use of Neogastropods as an Indicator of Tributyltin Contamination along the South China Coast. In: Newman MC (Ed.), Fundamentals of Ecotoxicology. CRC Press, Boca Raton, pp. 222–226.  Claremont, M., Vermeij, G.J., Williams, S.T., Reid, D.G., 2013. Global phylogeny and new classification of the Rapaninae (Gastropoda: Muricidae), dominant molluscan predators on tropical rocky seashores. Mol. Phylogenet. Evol. 66, 91–102.  Davies, I.M., Harding, M.J.C., Bailey, S.K., Shanks, A.M., Lange, R., 1997. Sublethal effects of tributyltin oxide on the dogwhelk *Nucella lapillus*. Mar. Ecol. Prog. Ser. 158, 191–204.  Fisher, W.S., Oliver, L.M., Walker, W.W., Manning, C.S., Lytle, T.F., 1999. Decreased resistance of eastern oysters (*Crassostrea virginica*) to a protozoan pathogen (*Perkinsus marinus*) after sublethal exposure to tributyltin oxide. Mar. Environ. Res. 47, 185–201.  Gibbs, P.E., Bryan, G.W., Pascoe, P.L., 1991. TBT-induced imposex in the dogwhelk, *Nucella lapillus*: Geographical uniformity of the response and effects. Mar. Environ. Res. 32, 79–87.  Gibbs, P.E., Bryan, G.W., Pascoe, P.L., Burt, G.R., 1987. The use of dog-whelk, *Nucella lapillus*, as an indicator of tributyltin (TBT) contamination. J. Mar. Biol. Assoc. UK 67, 507–523.  Horiguchi, T., 2006 Masculinization of female gastropod mollusks induced by organotin compounds, focusing on mechanism of actions of tributyltin and triphenyltin for development of imposex. Environ. Sci. 13, 77–87.  Horiguchi, T., Kojima, M., Kaya, M., Matsuo, T., Shiraishi, H., Morita, M., Adachi, Y., 2002. Tributyltin and triphenyltin induce spermatogenesis in ovary of female abalone, *Haliotis gigantea*. Mar. Environ. Res. 54, 679–684.  Horiguchi, T., Nishikawa, T., Ohta, Y., Shiraishi, H., 2007. Retinoid X receptor gene expression and protein content in tissues of the rock shell *Thais clavigera*. Aquat. Toxicol. 84, 379–388.  Horiguchi, T., Shiraishi, H., Shimizu, M., Morita, M., 1994. Imposex and organotin compounds in *Thais claviger*a and *T. bronni* in Japan. J. Mar. Biol. Assoc. UK 74, 651–669.  Horiguchi, T., Shiraishi, H., Shimizu, M., Morita, M., 1997. Imposex in sea snails, caused by organotin (tributyltin and triphenyltin) pollution in Japan: a survey. Appl. Organomet. Chem. 11, 451–455.  Horiguchi, T., Shiraishi, H., Shimizu, M., Yamazaki, S., Morita M., 1995. Imposex in Japanese gastropods (Neogastropoda and Mesogastropoda): effects of tributyltin and triphenyltin from antifouling paints. Mar. Pollut. Bull. 31, 402–405.  Horiguchi, T., Takiguchi, N., Cho, H.S., Kojima, M., Kaya, M., Shiraishi, H., Morita, M., Hirose, H., Shimizu, M., 2000. Ovo-testis and disturbed reproductive cycle in the giant abalone, *Haliotis madaka*: possible linkage with organotin contamination in a site of population decline. Mar. Environ. Res. 50, 223–229.  Huang, G.L., Yong, W., 1995. Effects of tributyltin chloride on marine bivalve mussels. Water. Res. 29, 1877–1884.  Hung, T.C., Hsu, W.K., Mang, P.J., Chuang, A., 2001. Organotins and imposex in the rock shell, *Thais clavigera*, from oyster mariculture areas in Taiwan. Environ. Pollut. 112, 145–152.  Kure, L.K., Depledge, M.H., 1994. Accumulation of organotin in *Littorina littorea* and *Mya arenaria* from Danish coastal waters. Environ. Pollut. 84, 149–157.  Leung, K.M.Y., Kwong, R.P.Y., Ng, W.C., Horiguchi, T., Qiu, J.W., Yang, R., Song, M., Jiang, G., Zheng, G.J., Lam, P.K.S., 2006. Ecological risk assessments of endocrine disrupting organotin compounds using marine neogastropods in Hong Kong. Chemosphere 65, 922–938.  Lyssimachou, A., Bachmann, J., Porte, C., 2008. Short-term exposure to the organotin compound triphenyltin modulates esterified steroid levels in females of *Marisa cornuarietis*. Aquat. Toxicol. 89, 129–135.  Morcillo, Y., Porte, C., 2000. Evidence of endocrine disruption in clams – *Ruditapes decussata* transplanted to a tributyltin-polluted environment. Environ. Pollut. 107, 47–52.  Oehlmann, J., 2004. Biological effects of contaminants use of intersex in the periwinkle (*Littorina littorea*) as a biomarker of tributyltin pollution. ICES Tech. Mar. Environ. Sci. 37, 22.  Oehlmann, J., Stroben, E., Schulte-Oehlmann, U., Bauer, B., 1998. Imposex development in response to TBT pollution in *Hinia incrassata* (Strom, 1768) (Prosobranchia, Stenoglossa). Aquat. Toxicol. 43, 239–260.  Okoro, H.K., Fatoki, O.S., Ximba, B.J., Adekola, F.A., Snyman, R.G., 2012. Development of an analytical method for determining tributyltin and triphenyltin in seawater, sediment, and mussel samples using GC-FPD and GC-MS-TOF. Pol. J. Environ. Stud. 21, 1743–1753.  Qiu, J.W., Chan, K.M., Leung, K.M.Y., 2011. Seasonal variations of imposex indices and butyltin concentrations in the rock shell *Thais clavigera* collected from Hong Kong waters. Mar. Pollut. Bull. 63, 482–488.  Roberts, M.H., Bender, M.E., DeLisle, P.F., 1987. Report (Project 9) for the Virginia State Water Control Board. Virginia Department of Environmental Quality, Richmond.  Ruiz, J.M., Barreiro, R., Gonzalez, J.J., 2005. Biomonitoring organotin pollution with gastropods and mussels. Mar. Ecol. Prog. Ser. 287, 169–176.  Salazar, M.H., Salazar, S.M., 1991. Assessing site-specific effects of TBT contamination with mussel growth rates. Mar. Pollut. Bull. 28, 621–628.  Schulte-Oehlmann, U., Oehlmann, J., Bauer, B., Fioroni, P., Leffler, U.S., 1998. Toxico-kinetic and -dynamic aspects of TBT-induced imposex in *Hydrobia ulvae* compared with intersex in *Littorina littorea* (Gastropoda, Prosobranchia). Hydrobiologia 378, 215–225.  Schulte-Oehlmann, U., Tillmann, M., Markert, B., Oehlmann, J., Watermann, B., Scherf, S., 2000. Effects of endocrine disruptors on prosobranch snails (Mollusca: Gastropoda) in the laboratory. Part II: triphenyltin as a xeno-androgen. Ecotoxicology 9, 399–412.  Shim, W.J., Kahng, S.H., Hong, S.H., Kim, N.S., Kim, S.K., Shim, J.H., 2000. Imposex in the rock shell, *Thais clavigera*, as evidence of organotin contamination in the marine environment of Korea. Mar. Environ. Res. 49, 435–451.  Sole, M., 2000. Effects of tributyltin on the MFO system of the clam *Ruditapes decussata*: a laboratory and field approach. Comp. Biochem. Physiol. C 125, 93–101.  Stenalt, E., Johansen, B., Lillienskjold, S.V., Hansen, B.W., 1998. Mesocosm study of *Mytilus edulis* larvae and postlarvae, including the settlement phase, exposed to a gradient of tributyltin. Ecotoxicol. Environ. Safe. 40, 212–225.  St-Jean, S.D., Pelletier, E., Courtenay, S.C., 2002. Hemocyte functions and bacterial clearance affected *in vivo* by TBT and DBT in the blue mussel *Mytilus edulis*. Mar. Ecol. Prog. Ser. 236, 163–178. |
| --- |
